# Survey of Tetrodotoxins (TTXs) in Gastropods, Sea Urchins, and Blue Crabs from the Adriatic Sea: First Report in *Paracentrotus lividus*

**DOI:** 10.3390/foods14234036

**Published:** 2025-11-25

**Authors:** Simone Bacchiocchi, Melania Siracusa, Giulia Diomedi, Simone Mazza, Erica Calandri, Tamara Tavoloni, Veronica Vivani, Monica Cangini, Giuseppe Arcangeli, Carmen Losasso, Silva Rubini, Gabriella Di Francesco, Francesca Leoni, Arianna Piersanti, Francesca Barchiesi

**Affiliations:** 1 Istituto Zooprofilattico Sperimentale Umbria e Marche “Togo Rosati”, Via G. Salvemini, 1, 06126 Perugia, Italy; s.bacchiocchi@izsum.it (S.B.); g.diomedi@izsum.it (G.D.); s.mazza@izsum.it (S.M.); e.calandri@izsum.it (E.C.); t.tavoloni@izsum.it (T.T.); a.piersanti@izsum.it (A.P.); 2 Centro di Referenza Nazionale per il Controllo Microbiologico e Chimico dei Molluschi Bivalvi Vivi, Istituto Zooprofilattico Sperimentale Umbria e Marche “Togo Rosati”, Via Cupa di Posatora, 3, 60131 Ancona, Italy; f.barchiesi@izsum.it; 3 Dipartimento di Scienze della Vita e dell’Ambiente, Università Politecnica delle Marche, Via Brecce Bianche, 60131 Ancona, Italy; v.vivani@pm.univpm.it; 4 University School for Advanced Studies IUSS, Piazza della Vittoria 15, 27100 Pavia, Italy; 5 National Reference Laboratory for Marine Biotoxins, Fondazione Centro Ricerche Marine, Viale A. Vespucci 2, 47042 Cesenatico, Italy; monica.cangini@centroricerchemarine.it; 6 Istituto Zooprofilattico Sperimentale delle Venezie, Viale Dell’Università 10, 35020 Legnaro, Italy; garcangeli@izsvenezie.it (G.A.); closasso@izsvenezie.it (C.L.); 7 Istituto Zooprofilattico Sperimentale della Lombardia e dell’Emilia-Romagna “Bruno Ubertini”, Via Modena 483, 44124 Cassana, Italy; silva.rubini@izsler.it; 8 Istituto Zooprofilattico Sperimentale dell’Abruzzo e del Molise “G. Caporale”, Via Campo Boario 1, 64100 Teramo, Italy; g.difrancesco@izs.it; 9 Laboratorio Nazionale di Riferimento Controllo Batteriologico Molluschi Bivalvi, Istituto Zooprofilattico Sperimentale Umbria e Marche “Togo Rosati”, Via Cupa di Posatora, 3, 60131 Ancona, Italy; f.leoni@izsum.it

**Keywords:** tetrodotoxin, survey, HILIC-MS/MS, sea urchins, gastropods, blue crab, Adriatic Sea, non-bivalve organisms, mussels

## Abstract

The detection of tetrodotoxins (TTXs) in European shellfish led the European Union to request a risk assessment from the European Food Safety Authority (EFSA). EFSA set a reference limit of 44 µg TTX equivalents kg^−1^ and called for more data on TTX occurrence, especially in gastropods, which can accumulate in TTXs but remain poorly studied. Recently, preliminary monitoring has revealed the recurrent presence of TTXs in mussels in three areas along the North–Central Adriatic coast of Italy, while research on non-bivalve organisms has not yet been carried out. This study presents a preliminary survey, conducted from January 2023 to March 2025, on the presence of TTXs in gastropods, echinoderms, and arthropods collected from this area. A method in Hydrophilic Interaction Liquid Chromatography coupled with tandem Mass Spectrometry (HILIC-MS/MS) for detecting TTXs in bivalve mollusks was first tested through an international proficiency test, then optimized for the other invertebrates, the object of this study. TTX levels in all gastropods and arthropod samples were undetectable, while traces (~5 µg kg^−1^) were found in one echinoderm sample (*Paracentrotus lividus*), marking the first reported occurrence of TTX in this species. Sea urchins are widely consumed in Italy; therefore, this finding is of particular importance from a public health perspective and deserves further investigation. Some gastropod genera or species sampled (e.g., *Nassarius*, *Rapana venosa*) have been identified as TTX carriers in other regions; therefore, the negative results obtained in this study may be related to seasonal or geographic variability. These results provide valuable data to EFSA’s call for monitoring emerging risks, particularly as climate change may increase TTX prevalence in European waters as well as worldwide.

## 1. Introduction

Tetrodotoxins (TTXs) are a group of extremely potent neurotoxins named after the fish family Tetraodontidae which includes most of the pufferfish species. The guanidinium group in the molecule allows it to bind to voltage-gated sodium channels on muscle and neuronal cell membranes. This interaction blocks nerve signal transmission by preventing sodium ion influx through the channels [1,2,3,4,5,6]. The consumption of TTX-contaminated seafood quickly induces a wide range of symptoms, ranging from mild numbness of the mouth and tongue to paralysis or even death. Beyond pufferfish, TTXs have been detected in numerous other marine organisms including Tetraodontidae, Diodontidae, and Molidae, as well as in bivalve mollusks, gastropods (sea slugs), echinoderms, crabs, and ribbon worms [7,8,9,10,11,12,13]. One of the reasons for the spread of TTXs in many marine species, even phylogenetically distant and at different levels of the food chains, could be their supposed bacterial origin. *Vibrio*, *Pseudomonas*, *Bacillus*, *Actinomyces*, and *Micrococcus* are the genera usually recognized as TTX producers [14,15,16,17]. These bacteria are widely distributed in marine environments and enter multiple trophic chains. In many cases, the TTX-producing strains were initially isolated from TTX-contaminated marine organisms [14].

Historically, TTXs posed significant health concerns, primarily in the Indo-Pacific region, especially in countries like Japan, Thailand, Bangladesh, and Philippines, where pufferfish (*Fugu*) is consumed as a traditional culinary delicacy, resulting in dozens of deaths each year [18]. However, recent decades have witnessed an expansion of TTX into new geographical areas, including the Eastern Mediterranean and various parts of Europe. One of the main drivers of this phenomenon is certainly the climate changes currently occurring at a global level, which have caused TTX-bearing species, such as pufferfish, to settle in higher-latitude waters and have created particularly favorable conditions for the proliferation of TTX-producing bacteria, such as *Vibrio*. For this reason, EFSA has identified TTXs as one of the emerging threats to human health in the future. Since 2003, reports of poisonous pufferfish entering the Mediterranean via the Gulf of Suez have increased, along with incidents of poisoning from its consumption, primarily in the eastern part of the basin (including Crete, Lebanon, Israel, Libya, and Turkey), with a gradual spread toward the west [19,20,21,22,23,24,25]. Starting from 2015, TTXs have been detected in bivalve mollusks from various European countries (the United Kingdom, Spain, Greece, the Netherlands, Portugal, and Italy) [26,27,28,29,30,31,32,33], and in 2017, in response to this emerging threat, the European Food Safety Authority (EFSA) published a scientific opinion proposing a provisional safety threshold of 44 µg TTX equivalents per kilogram of shellfish meat [34]. In the EFSA opinion, the need to acquire data on the occurrence of TTXs in gastropods is emphasized by asking member states for monitoring. According to the literature, gastropods are mollusks able to accumulate TTXs, reaching contamination levels up to 1000 times higher than those found in bivalves [35], and can cause intoxication in humans. Nevertheless, bivalve mollusks have been the subject of a much larger number of studies, mainly because they are included in official food safety monitoring programs. Gastropods, on the other hand, have been investigated less frequently.

Recently, blue crab (*Callinectes sapidus*), a native species of the Atlantic coasts of Canada, North, and South America, probably driven by the ongoing climate changes, and as a result of its ecological adaptability, invaded the Northern Adriatic Sea [36], causing a dramatic impact on the local shellfish farming industry, as a result of its voracity towards bivalve mollusks and its high fecundity in absence of natural predators. One of the proposed actions to mitigate the invasion is the promotion of its consumption. However, the distribution of blue crab as food needs a thorough risk assessment of the potential hazards to which consumers may be exposed. Blue crabs inhabit estuaries, tidal marshes, and coastal lagoons, feeding on small organisms such as mollusks, fish, and detritus. In the Northern Adriatic Coasts, there are numerous lagoons hosting bivalve mollusk farming and harvesting industry. The Marano Lagoon (Friuli–Venezia Giulia Region) has recently been identified as a hotspot of TTX accumulation in mussels [32] and TTX mussel contamination was also reported in 2023 and 2024 in Emilia-Romagna [37]. The predation of TTX-contaminated mollusks by blue crabs could represent a pathway for biomagnification of these toxins along the food chain. Also, the Conero Riviera (Marche Region) has been identified as a hotspot, where TTXs periodically accumulate in mussels and other biotic matrices [33,38], posing a possible risk to consumers’ safety. Therefore, it would also be advisable to investigate the distribution of TTXs in the different autochthonous organisms, particularly those used as food, in the latter area.

Liquid Chromatography coupled with tandem Mass Spectrometry (LC–MS/MS) is considered the gold standard for the detection and quantification of marine biotoxins, because of its ability at providing direct structural identification of toxins, although particularly susceptible to the matrix effect. Also, biological or immune assay-based methods are used in the field of biotoxin monitoring but with some drawbacks (no unequivocal structural identification of the analytes, no analogue selectivity, general overestimation of the concentrations, etc.).

This work aims to conduct a preliminary survey on TTXs in various marine organisms, other than mussels, harvested in the Northern and Central Adriatic Sea during 2023–2025. Roughly 100 samples belonging to eight different genera and species of gastropods, 30 samples of blue crab, coming mainly from the transitional coastal areas of the North–Central Adriatic Sea and more than 20 samples of echinoderms from the Conero Riviera were analyzed in order to assess the potential TTX risk associated with seafood consumption and meet EFSA’s request of increasing the data availability on TTXs in gastropods.

All the studied species are edible, but human consumption varies considerably among them. *Bolinus brandaris*, *Nassarius mutabilis*, *Hexaplex trunculus*, *Patella caerulea*, *Brachyura* spp., *Maja squinado*, and *Squilla mantis* are widely consumed throughout the Mediterranean area. *Paracentrotus lividus* is mostly regarded as delicacy, particularly within the Italian culinary tradition. *Rapana venosa* is highly appreciated in Japan and Korea, whereas *Galeodea echinophora*, *Aporrhais pespelecani*, and *Tonna galea* are mainly collected for ornamental purposes. *Callinectes sapidus* is an alien species that is now widespread in the Mediterranean Sea, and its consumption has recently been increasing.

To achieve the monitoring objective, an LC-MS/MS method was developed, optimized, and validated in the considered matrices.

## 2. Materials and Methods

### 2.1. Sampling Design

**Figure 1:**
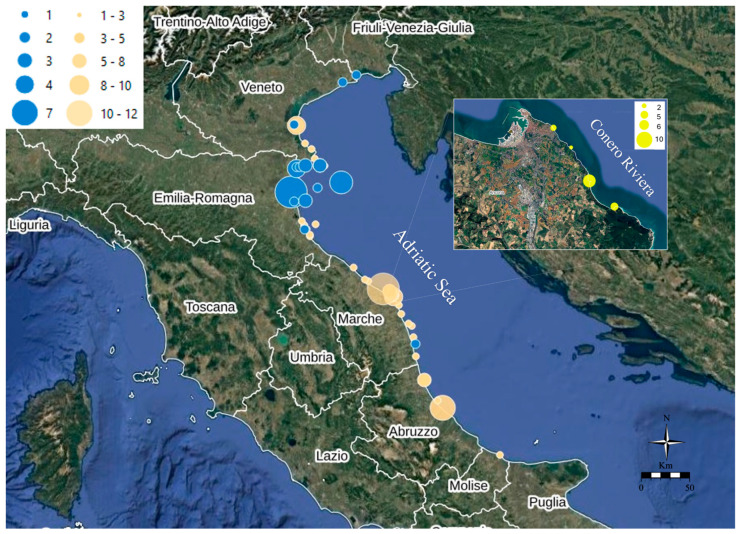
Gastropods (peach), arthropods (blue), and sea urchins (yellow) sampling sites along the North–Central Adriatic Sea coasts of Italy. Circular markers of increasing size illustrate the number of samples collected at each site. Images (data SIO, NOAA, U.S. Navy, NGA, GEBCO; image Landsat/Copernicus) are from Google Earth.

The sampling campaign was conducted in the frame of the research project “IZSUM 02/22 RC”, funded by the Italian Ministry of Health, by the network of the Italian Istituto Zooprofilattici Sperimentali including the Istituto Zooprofilattico Sperimentale dell’Umbria e delle Marche (IZSUM), delle Venezie (IZSVe), della Lombardia ed Emilia-Romagna (IZLER), dell’Abruzzo e Molise (IZSAM) in collaboration with the Università Politecnica delle Marche (UNIVPM), and the National Reference Laboratory for Marine Biotoxins. Gastropods, echinoderms, and arthropods were collected from the North–Central Adriatic Sea coast of Italy (FAO area 37.2.1), specifically in Friuli–Venezia Giulia, Veneto, Emilia-Romagna, Marche, Abruzzo, and Molise regions (Figure 1). The sampling area spans approximately 700 km in length, the sea has a depth ranging from 15 to 35 m in the Northern Adriatic and from 50 to 70 m in the Central Adriatic, and the seabed features a wide variety of morphologies and different substrates: the coasts are predominantly sandy and muddy, with the presence of rocky zones and lagoon areas. The salinity ranges between 38.4‰ and 38.9‰ [39] with a decreasing trend towards north, as a result of the Po River influence. The Adriatic basin, narrow and elongated in the latitudinal direction, exhibits marked seasonal variability with an average annual temperature of 17.5 °C. Marine currents are weak and flow counterclockwise from the Strait of Otranto northward along the Croatian coast, and southward on the Italian coast. The Conero Riviera is located in Central Italy in the province of Ancona (Marche region), and features the unique characteristics of a high, jagged, and rocky coastline. Here, bivalve mollusks, gastropods, and echinoderms live attached to the submerged rocks within complex biocoenoses.

**Figure 2:**
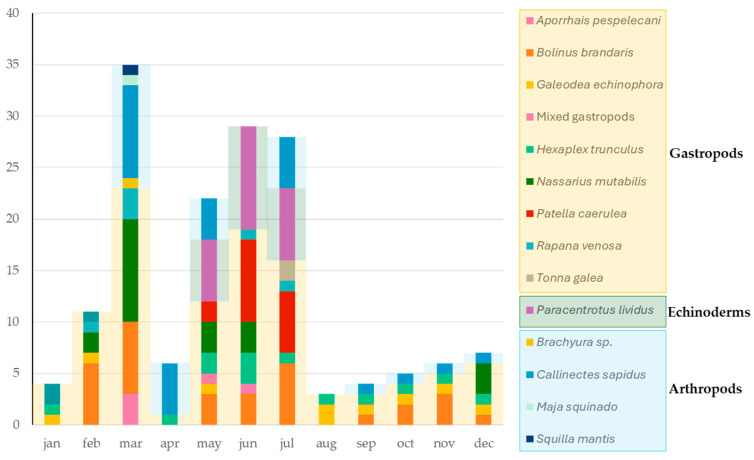
Gastropods, echinoderms and arthropods species collected during the sampling campaign conducted in the North–Central Adriatic Sea between 2023 and 2025.

The experimental design foresees the collection of at least three gastropod samples per month, throughout the entire study period (2023–2025—27 months), and at least one sample of *C. sapidus* per month in the northern regions. An increase in number was recommended in the period included between March and July, because of the known seasonality of TTX accumulation. Special attention was devoted to the Conero Riviera [38], identified as TTX hotspots in the North–Central Adriatic Sea, during the months of May–July, collecting, bi-weekly, *P. lividus* and *P. caerulea* (Table 1 and Table S1, Figure 1 and Figure 2).

Since gastropods, echinoderms, and arthropods are not subject to regular monitoring by the competent authorities, the number of samples was not exactly reproducible.

A total of 104 samples of marine gastropods belonging to 8 different genera and species including the purple dye-murex (*B. brandaris*), the banded dye-murex (*H. trunculus*), the helmet shell (*G. echinophora*), the giant tun (*T. galea*), the rapa whelk (*R. venosa*), the nassa snail (*N. mutabilis*), the pelican’s foot (*A. pespelecani*) and blue limpet (*P. caerulea*), and 33 samples of arthropods, including 30 samples of blue crab (*C. sapidus*), one of true crab (*Brachyura* spp.), one of European spider crab (*M. squinado*), and one of mantis shrimp (*S. mantis*), were harvested from January 2023 until March 2025 throughout the whole North–Central Adriatic Sea. A total of 23 sea urchins (*P. lividus*) were collected from the Conero Riviera during May–July in 2023 and 2024 (Table 1 and Table S1, Figure 1 and Figure 2, and Figure S1).

Different sample sizes (number of specimens) were adopted for the various species collected in order to obtain representativeness. The number of individuals was determined based on the animal size and on the amount of the specimen available tissue. Regarding the analyzed sample, pools of specimens were used, as recommended by the Standard Operating Procedures for the Determination of Marine Biotoxins of the EU Reference Laboratory for Monitoring of Marine Biotoxins (EURLMB) (https://www.aesan.gob.es/en/CRLMB/web/public_documents/seccion/crlmb_standard_operating_procedures.htm, accessed on 19 November 2025). The number of organisms pooled in each sample was chosen to ensure analytical representativeness, as described in Section 2.2.

### 2.2. Sample Treatment

Gastropod samples were externally washed, and the shell was broken using a hammer in order to collect the entire specimen soft tissue, including digestive gland and gonads. Shell fragments and the operculum were then removed. The sea urchin shell was opened with scissors and the flesh collected. Limpet samples were processed by separating the body from the shell with scalpel and scissors and collecting the tissue. Regarding crab samples, the dorsal carapace was removed using scalpel and scissors, and the internal tissues collected. Additionally, the muscle from inside the claws was sampled.

All tissues were placed on a mesh screen to drain off excess saltwater. To ensure representativeness, at least 100–150 g of pooled flesh was collected, homogenized, and stored at −20 °C until chemical analysis.

### 2.3. Chemical Analysis of TTXs

#### 2.3.1. Chemicals and Standards

All reagents used were of at least analytical grade. Acetonitrile (LC-MS grade) and methanol (HPLC grade) were purchased from CARLO ERBA Reagents S.r.l. (Cornaredo, Milan, Italy). Ammonium hydroxide (≥25% in water, LC-MS grade) was obtained from Merck (Darmstadt, Germany), formic acid (LC-MS grade) from VWR International (Radnor, PA, USA), and glacial acetic acid (reagent grade) from Sigma-Aldrich (Steinheim, Germany). Ultrapure water was produced using a Milli-Q water purification system (Millipore Ltd., Bedford, MA, USA).

The certified reference material (CRM) for tetrodotoxin (CRM-03-TTXs) was supplied by CIFGA Laboratory (Lugo, Spain): a mixture containing tetrodotoxin (25.9 ± 1.3 µg g^−1^) and 4,9-anhydro tetrodotoxin (2.99 ± 0.16 µg g^−1^).

The stock solution was prepared in a solution of acetonitrile (80% *v*/*v*) containing acetic acid (0.25% *v*/*v*) starting from the CRM. Matrix-matched calibration standards were obtained by diluting the stock solution in extracts of a blank sample of the matrices included in the study. The stock solution was also used to prepare samples for quality control and method validation.

#### 2.3.2. TTX Extraction

The TTX analytical method developed comes from the fusion of two EURLMB official protocols: the “EURLMB SOP for the analysis of Paralytic Shellfish Toxins (PST) by precolumn HPLC-FLD according to OMA AOAC 2005.06” (EURLMB SOP for PST) [40] for the extraction of mussels, gastropods, echinoderms, and arthropods and the EURLMB SOP “Determination of Tetrodotoxin by HILIC-MS/MS” (EURLMB SOP for TTX) [41] for the protein precipitation step and the final dilution applied to reduce the matrix effect, as already described by Bacchiocchi et al [38].

The extraction protocol of the EURLMB SOP for PST was chosen because it ensures an accurate final extract volume and then the same dilution factor for all samples, and it harmonizes different analytical protocols suitable for the determinations of Paralytic Shellfish Toxins (PST) and TTXs. Instead, the EURLMB SOP for TTX was adopted in the cleaning step, because this approach to reducing matrix effects does not involve significant dilution. A schematic diagram of the protocol fusion is reported in Figure S2.

Briefly, 5.0 ± 0.1 g of flesh homogenate was extracted with 3 mL of acetic acid 1% (*v*/*v*), vortex-mixed for 2 min, and placed in a boiling water bath (100 °C) for 5 min. The extract was cooled to room temperature, vortex-mixed for 2 min, and centrifuged at 3220× *g* for 10 min. The supernatant was collected, and the solid residue re-extracted a second time in the same conditions overlooking the boiling step. The combined extracts were then brought to 10 mL with water. Ammonium hydroxide (5 µL) was added to the supernatant (1 mL), the sample was then vortex-mixed for 3 min and centrifuged at 10,000× *g* per 1 min. The final extract was diluted (1:2) with a solution of acetonitrile (80% *v*/*v*) containing acetic acid (0.25% *v*/*v*), filtered through a 0.2 µm syringe filter and analyzed by Hydrophilic Interaction Liquid Chromatography coupled with tandem Mass Spectrometry (HILIC-MS/MS).

#### 2.3.3. HILIC-MS/MS Analysis

HILIC-MS/MS analysis was carried out using the same conditions already reported by Bacchiocchi et al. [38]. The chromatographic separation was carried out following the EULMB SOP “Determination of Tetrodotoxin by HILIC-MS/MS” [41] under the analytical conditions described in Table S2. Instrumental analysis was performed using an ACQUITY I-Class–Xevo TQ-S micro IVD system (Waters, Milford, MA, USA), equipped with an electrospray ionization (ESI) source operated in positive ion mode. A total of nine TTX analogs were monitored in multiple reaction monitoring (MRM), selecting two transitions each, to ensure accurate identification and quantification as described in Table S2. A chromatogram of a matrix-matched standard solution (2 µg mL^−1^) containing the analogues TTX, 4,9-anhydro tetrodotoxin (4,9-anhydro TTX), and the non-certified analogue 4-epi tetrodotoxin (4-epi TTX) is reported in Figure S3. The TTX identification in the samples was based on retention times and ion ratio comparison between samples and a matrix-matched standard. Certified reference standards are not commercially available for all the analogues; therefore, for those analytes lacking reference, the identification was based on specific MRM transitions reported in the literature. All analogs were quantified using TTX CRM, assuming an equimolar response.

#### 2.3.4. HILIC-MS/MS Method Validation/Optimization

The method reported by Bacchiocchi et al. was in-house validated in mussels, testing linearity at six concentrations (6.5–130 ng mL^−1^) via matrix-matched curves, limit of detection (LOD), limit of quantification (LOQ), accuracy (R%), and precision in terms of intraday repeatability (RSDr%) at 75 and 251 µg kg^−1^ [33,38]. The reported method performances were obtained on TTX, being the main analogue present in the naturally contaminated mussel samples, and the only for which CRM is available. The 4,9-anhydro TTX listed in the certificate is present at very low concentrations, not enabling its use for full validation. The lack of CRMs for TTX analogues is a well-known issue in the field of marine biotoxins, forcing analysts to find alternative approaches to test the performances for other analytes included in the method [42].

The HILIC-MS/MS method was further tested for its performances and applicability participating, in January 2023, to the international Proficiency Test (PT) organized by WEPAL-QUASIMEME (Wageningen University—Netherlands—DE16—Tetrodotoxin in shellfish, 2022, round 2) together with 13 other laboratories from across Europe. The PT results were evaluated in terms of Z-score which indicates how far the laboratory’s results are from the assigned value considering the provided standard deviation.

Then, method performances at lower contamination levels for mussels (8 and 22 µg kg^−1^ in stand of 75 and 251 µg kg^−1^) and its applicability to the other matrices (gastropods, echinoderms, arthropods) were investigated.

Specific effort was put into studying the matrix effect and the matrix equivalence. Matrix effect was evaluated comparing the slopes of the calibration curves built in different matrices, using blank extracts (previously analyzed and confirmed to be free of TTXs above the quantification limit) of mussels (*Mytilus galloprovincialis*), echinoderms (*P. lividus*), gastropods (*N. mutabilis*), and arthropods (*C. sapidus*), and that obtained in solvent (acetonitrile/water 80:20, v/v, containing 0.25% acetic acid) [43].

Each curve included seven concentration levels (0, 1.99, 3.98, 8.09, 16.19, 32.38, and 64.75 ng mL^−1^) interpolated by linear regression using the least squares method. All the analyses were conducted in triplicate, on three different days. The coefficient of determination (R^2^) was calculated for each calibration curve to assess linearity. Matrix effect was measured calculating the percentage ratio between the calibration curves’ average slopes obtained for each matrix (mussels, echinoderms, gastropods, and crustaceans) and the slopes of the solvent calibration curves. The matrix effect was considered negligible when this value fell within the range of 80–120% because this variation is close to the repeatability values [43]. Moreover, also the matrix equivalence among the species was considered, comparing the percentage ratio of the calibration curves’ average slopes for echinoderms, gastropods, and blue crabs with that of mussels. The studied matrices were considered equivalent to mussels if the ratio fell within the range 80–120%.

Matrix equivalence among the species also for 4,9-anhydro TTX (present in the standard solution at a concentration of 1/9 the TTX) was evaluated using the two highest calibration levels of the three curves, corresponding to 3.73 ng/mL and 7.45 ng/mL, respectively. The percentage area ratios for echinoderms, gastropods, and blue crabs with respect to mussels were compared using the same criteria described above.

The TTX LOQ for bivalve mollusks was determined by Bacchiocchi et al. [38] estimating the concentration corresponding to a signal-to-noise (S/N) ratio of 10. The LOQ was then experimentally confirmed through repeated analysis of spiked samples. The LOD was calculated by dividing the experimental LOQ by 3.3. The resulting LOD and LOQ values of 3 and 8 µg kg^−1^, respectively [38], were extended to all matrices considered in the study and the LOQ was experimentally verified by fortifying blank samples.

Replicated analysis on mussel samples spiked with TTX at 8 and 22 µg kg^−1^ was performed. The applicability to the other matrices (gastropods, echinoderms, arthropods) was also tested by repeated analysis of spiked gastropods and echinoderms (8, 22 µg kg^−1^). In the case of arthropods, 16 and 32 µg kg^−1^ TTX concentrations were tested. Duplicated analyses of each concentration level were performed on six different days.

Accuracy (R%) and precision (inter-day relative standard deviation RSD_R_%) were evaluated in all the considered matrices at the two concentration levels. The quantification was carried out using a mussel matrix-matched calibration curve.

Internal quality controls were included in each analytical batch by spiking a blank mussel with TTX at the LOQ. Accuracy in terms of R% was calculated to check validation performances. In cases of samples with ambiguous interpretation, co-chromatography was performed, spiking it with a comparable amount of TTX, to unequivocally identify the analyte.

Concentrations falling between LOD and LOQ were reported as estimated values, with the awareness that they carry greater uncertainty than those >LOQ.

#### 2.3.5. Statistical Analysis

The significance of the slope differences was evaluated using a t-test (*p* < 0.05) (Stata^®^ 16.1 Special Edition; StataCorp LP, College Station, TX, USA) acquiring ten calibration curves at the seven concentration levels previously listed, for each matrix (echinoderms, gastropods, blue crabs and mussels), on five different days.

## 3. Results and Discussion

### 3.1. Method Performances

The results obtained by the IZSUM’s laboratory in the WEPAL-QUASIMEME PT were satisfactory, with a Z-score well below 2 for the two contaminated samples distributed by the organization: a mollusk homogenate (QTT014BT) and a standard solution (QTT012SS), indicating high analytical accuracy. Furthermore, the uncontaminated mollusk homogenate sample (QTT013BT) was correctly classified as “not detectable,” confirming the specificity of the method (Table 2).

Regarding the study of the matrix effect, the calculation of the percentage ratio between the average calibration curve slopes revealed a marked difference between the solvent and the four analyzed matrices (mussels, echinoderms, gastropods, and blue crabs), indicating a significant signal suppression in the presence of matrices. In contrast, the comparison among the different matrices showed similar slopes, suggesting similar behaviors and, consequently, substantial matrix equivalence among the species considered (Figure S4). The percentage ratios (% ratio) between the matrix-matched curve slopes and the slope of the solvent curve or the slope of the mussel matrix-matched curve are reported in Table 3. Matrix signal suppression in the studied species ranged between 70% and 71% compared to the solvent.

In the case of matrix equivalence, the percentage ratios were all within the acceptable range of ±20%. Moreover, the t-test (*p* < 0.05) did not show significant differences between the slopes obtained for mussels and the other matrices included in the study. These results confirmed the matrix equivalence among the species considered.

Regarding the 4,9-anhydro TTX, the percentage ratios were all within the acceptable range of ±20% (Table S3), underlining the matrix equivalence also for this analogue.

Regarding LOD and LOQ, experiments highlighted that echinoderm and gastropod matrices behave similarly and were confirmed to be equal to 3 µg kg^−1^ and 8 µg kg^−1^, respectively. Conversely, for blue crab, the achievable LOD and LOQ were higher, reaching 5 and 16 µg kg^−1^, respectively. Table S4 summarizes the results obtained for all relevant parameters studied, for each tested level and matrix: the single measured concentrations (µg kg^−1^) and their average values (µg kg^−1^) with the corresponding standard deviations (SD), the recoveries (R%), their average values with the corresponding standard deviation (SD), and the precision reported as RSD_R_%. The recoveries obtained for echinoderms and gastropods which fell within the accuracy range defined for mussels was 76 ± 18% at 8 µg kg^−1^ and 92 ± 20% at 22 µg kg^−1^ level. The spiking levels tested for blue crab were both higher in respect to the other matrices considered (16 and 32 µg kg^−1^) due to higher fat-soluble pigment content compared to mussel, which results in greater matrix suppression [44], but when comparing the obtained results with the ones of mussels spiked at 22 µg kg^−1^, similar results were observed in terms of R%. Also, the precision, expressed in terms of RSD_R_% was comparable to or lower than that observed in mussels.

Therefore, the results of the robustness study performed on the method developed, validated, and tested on bivalve mollusks have demonstrated its applicability also to echinoderms, gastropods, and arthropods with good performances. Moreover, the decision to omit the clean-up step proposed in the EURLMB SOP for TTX proved advantageous, as it allowed the achievement of sufficiently low LOQs across all tested matrices. Given that the LC-MS/MS system used is classified as a medium-sensitivity instrument [45], including the clean-up step would have introduced a significant dilution factor, thereby compromised method sensitivity and preventing the attainment of low LOQs, as previously demonstrated in the literature [42].

Considering these results, the developed method makes it possible to achieve the best performance in terms of sensitivity, robustness, and accuracy across a wide range of marine invertebrates by using equipment accessible to most laboratories responsible for monitoring marine biotoxins. Furthermore, this type of approach allows for significant savings in analytical time (protocols simplified and shared with those for other marine biotoxins) and costs (reduced use of analytical standards and clean up devices).

### 3.2. TTXs Survey in North–Central Adriatic Sea

The EFSA opinion emphasized the need for additional data on the occurrence of TTXs in bivalves and gastropods, as well as further investigation on their origin and trophic transfer [34]. Furthermore, according to the EFSA report from the CLEFSA Project (CLimate change and Emerging risks for Food SAfety) published in 2020, TTXs are among the biotoxins expected to increase significantly in Europe as a result of climate change [46] and are therefore considered to be among the main emerging public health risks. Since 2017, preliminary monitoring has been carried out along the North–Central Adriatic coast of Italy to assess the presence of TTXs in bivalve mollusks. These studies revealed exceptionally high toxin levels (up to 541 µg kg^−1^), the highest ever found in Europe, in mussels from the Marano Lagoon, in Friuli–Venezia Giulia [32], mussels exceeding the EFSA alert threshold (up to 296 µg kg^−1^) in the Conero Riviera (Marche) [38] and detectable contamination in mussels from Marina di Ravenna (Emilia-Romagna) [37]. Thus far, no research has been undertaken on gastropods, echinoderms, arthropods, or other marine organisms in these regions. In order to contribute to a proper risk assessment of TTX contamination in seafood, within the present work, an extended survey was carried out on gastropods, echinoderms, and arthropods from various areas of the North–Central Adriatic Sea.

Gastropods, after pufferfish, are the second most common source of TTX human intoxication worldwide, due to the consumption of contaminated seafood, with the vast majority of cases related to the *Nassarius* genus [35]. So far, as many as 13 *Nassarius* species were reported to be contaminated by TTXs, mainly from China, Japan, and South Korea [47], with levels up to 2.1 g kg^−1^ in *N. glans* [48], among the highest values ever recorded in marine organisms after pufferfish. Also, other gastropod genera have been found to harbor TTXs: *Gibbula*, *Monodonta*, *Charonia*, *Patella*, *Nucella*, *Onchidella*, and *Aplysia* in Portugal [11,49], *Pleurobranchaea* in New Zealand [9], *Rapana, Natica*, *Polinices*, *Oliva* in Taiwan [50,51,52], *Rapana*, *Bullacta,* and *Neverita* in China [53], *Buccinum* in France [54], *Babylonia* and *Tutufa* in Japan [55,56], *Onchidella* in Morocco [11], and *Umbonium* from Taiwan [57]. Significant concern was raised by the only documented case in Europe of severe human poisoning associated with the consumption of an autochthonous species in 2007. A person in Spain ingested trumpet shell meat (*Charonia lampas*), caught off the southern coast of Portugal, containing 315 mg kg^−1^ of TTXs [30]. The present study makes quite extensive monitoring of gastropods, including a total of 104 samples belonging to eight different genera and species (Table 1), for which TTX levels were always below the LOD, for all the analogues investigated (Table S1). This suggests a low accumulation capacity of the organisms collected in North–Central Adriatic Sea despite the sampling season; in fact, the campaign was mainly undergone in the months of the year known to be critical for TTX accumulation in mussels. It is to be highlighted that the gastropods sampling campaign, in certain specific areas like the Conero Riviera, was conducted in the same period in which mussels (*M. galloprovincialis*) were also collected. The latter showed measurable levels of TTX ranging from 3 to 119 µg kg^−1^ [58].

**Figure 3:**
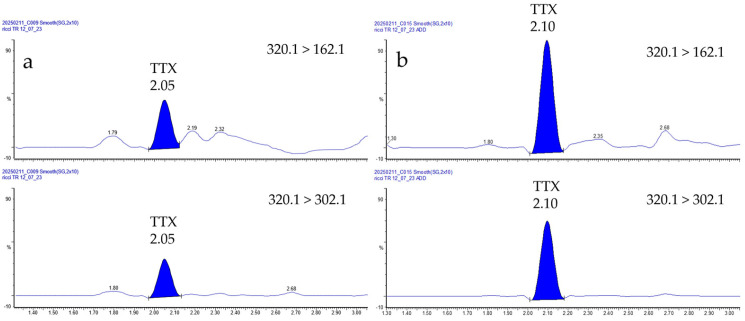
HILIC-MS/MS chromatograms of the (**a**) sea urchin (*P. lividus*) sample collected from the Conero Riviera on 12 July 2023 (~5 µg kg^−1^); (**b**) same sample spiked with 10 µg kg^−1^. 320.1 > 162.1, multiple reaction monitoring (MRM) transition for TTX quantification; 320.1 > 302.1, MRM transition for TTX confirmation.

This relevant result showed that, despite sharing the same environments, different organisms do not exhibit unique behaviors in TTX accumulation. Among the species analyzed, *B. brandaris*, *H. trunculus*, *G. echinophora*, *T. galea*, and *R. venosa* live in the Mediterranean Sea: the first two are widely distributed along the coasts from Gibraltar towards eastern Mediterranean while *G. echinophora* and *T. galea* exhibit a patchier distribution. *R. venosa* is an alien species introduced into the Mediterranean from the Western Pacific [59]. All of them prefer shallow, sandy, or muddy seabeds at depths ranging from 5 to 50 m (up to 120 m for *T. galea*), and are voracious predators of cnidarians, echinoderms, mussels, and clams. In the Adriatic Sea, mussels have been found to be contaminated with TTX [32,37,38], therefore yielding to a possible biomagnification in predator species. *N. mutabilis* and *A. pespelecani* are widely distributed throughout the Mediterranean Sea, including Adriatic. They thrive in estuaries and lagoons, where organic matter is abundant; they are scavengers, burrowing into the sediment to feed on organic debris. These species could accumulate TTXs from sediment, especially in the lagoons of the Northern Adriatic, where the circulation of these toxins has been demonstrated [32,37]. *P. caerulea* is found in the Mediterranean Sea and the eastern Atlantic Ocean. It inhabits rocks in the intertidal zone and feeds on encrusting algae and bacteria biofilm, from which it may accumulate TTXs. Although, the *Nassarius*, *Patella* genera and the *R. venosa* species have been described as capable of accumulating TTXs [11,35,52], in the present study, none of the 21, 16, and 6 respective samples (Table 1) showed levels above the LOQ. This could suggest a lower accumulation capacity of the organisms collected in the North–Central Adriatic Sea compared to other gastropods or the effect of the habitat and of the season considered, not promoting TTX accumulation. Surely, the limited number of samples and the short period of time considered could have affected the results. *N. mutabilis* is widely consumed in the Italian diet, and considering the TTX accumulation capacity reported for the *Nassarius* genus, even at potentially lethal levels, a more extensive monitoring effort may indeed be necessary. Regarding sea urchins, TTXs were reported at levels above the EFSA safe threshold in *Arachnoides zelandiae* in North–Western New Zealand [60] and at nearly negligible levels in *Echinus esculentus* in Portugal [48]. Sea urchins are omnivorous benthic grazers, feeding on algae, detritus, and small invertebrates, and TTXs can accumulate in them through the ingestion of contaminated prey or biofilms harboring TTX-producing bacteria. All the samples of *P. lividus* analyzed in the present study (Table 1) were collected between May and July 2023–2024 in the Conero Riviera, a specific hotspot where TTX accumulation in mussels and in other benthic species is a phenomenon already observed in this specific period [58]. Almost all the 23 samples showed non-detectable TTX levels for all analogues investigated (Table S1), consistent with the previously reported literature data for *P. lividus* in Portugal [11]. Only one sample, collected on 12 July 2023, showed traces of TTX (~5 µg kg^−1^, Figure 3, Table S1). To the best of our knowledge, this is the first report of TTXs in this sea urchin species (*P. lividus*) worldwide. We recognize that sea urchin is consumed mostly as delicatessen in the frame of regional traditions, but in Italy, especially in the South, it is deeply rooted in the culinary culture, and its consumption has been constantly increasing. To date, it exceeds 2000 tons per year [61], making Italy one of the largest consumers in Europe. In recent years, the harvesting of *P. lividus* has become increasingly restricted and regulated due to the need to protect the species from overfishing and possible extinction. Therefore, a proper risk assessment is surely recommended.

Although the consumption of contaminated arthropods ranks third in terms of the percentage of human cases of TTX poisoning, the only species linked to intoxication episodes is the horseshoe crab *Carcinoscorpius rotundicauda* [35]. However, some Xanthid crabs, *Zosimus aeneus*, *Atergatis floridus* in Japan [62,63], *Lophozozymus Pictor* and *Demania reynaudi* in Taiwan [63,64], *Demania cultripes* in the Philippines [65], and the edible crabs *Afruca tangeri* and *Carcinus maenas* from Portugal [66] have also proven to be TTXs bearers.

The blue crab (*C. sapidus*), especially the male, spends a large part of its life cycle near estuaries and lagoons in brackish waters. In the North–Western Adriatic coasts (Friuli–Venezia Giulia, Veneto, and Emilia-Romagna, Italy), it shares the habitat with bivalve mollusks (mussels and clams), on which it preys with voracity. In the Marano Lagoon (Friuli–Venezia Giulia) and in Marina di Ravenna mussel farms (Emilia-Romagna), the presence of TTXs in mussels has been periodically and/or sporadically reported in recent years [32]. The present work wanted to investigate the potential transfer of TTXs from bivalve mollusks to blue crabs. All 30 blue crabs analyzed, as well as the three single samples of *Brachyura* spp., *M. squinado*, and *S. mantis*, which were collected almost exclusively in the Northern Adriatic Sea (Figure 1, Table 1), showed TTX levels below the LOD for all investigated analogues (Table S1). This finding suggests either a low capacity of these species to accumulate TTX or that the sampling periods and/or geographic locations were not favorable to such accumulation. As far as we know, no other studies exist on TTXs in *C. sapidus* to compare the results obtained; therefore, from the data collected to date, we can state that it does not accumulate TTXs. However, given the limited sample size, further studies on a larger population and extended observation periods are needed to support or rebut this preliminary result.

## 4. Conclusions

The HILIC-MS/MS method developed and validated in the present study demonstrated high analytical accuracy and specificity for the determination of TTXs in bivalve mollusks enabling them to reach adequate LOQs, as confirmed by successful participation in an international proficiency test. Moreover, the applicability of the proposed protocol to other taxonomic groups, namely gastropods, echinoderms, and arthropods was demonstrated showing matrix equivalence, also with mussels, thus allowing TTX monitoring in a wider spectrum of marine organisms. The preliminary survey conducted in the North–Central Adriatic Sea on 104 samples of gastropods, 23 sea urchins, and 33 arthropods revealed non-detectable TTX levels in all the analyzed samples, except for only the *P. lividus* sample which showed traces of TTX (~5 µg kg^−1^). This suggests a limited ability of the species investigated to accumulate TTXs, or a low exposure to TTX sources during the sampling time considered. Nevertheless, the presence of known TTX-accumulating species among those studied, combined with some ecological traits and dietary relevance, highlights the need for further investigation. Considering the increasing attention to TTXs as an emerging food safety risk in Europe in light of climate change-driven toxin distribution modification, these findings contribute to a better understanding of TTX occurrence in non-bivalve seafood species. Further data are needed for comprehensive risk assessment.

## Figures and Tables

**Table 1:** Samples collected in North–Central Adriatic Sea between 2023 and 2025. Specimens per sample and pooled specimens for LC-MS/MS analysis.

Phylum	Species	N° of Samples	Sample Size (Specimens per Sample)	Sampling Area *	Sampling Period	Analyzed Sample (Pooled Specimens)
Mollusks (Gastropods)	*Bolinus brandaris*	32	50	Ve, ER, Ma, Ab	January 2023– March 2025	15
	*Nassarius mutabilis*	21	100	ER, Ma, Ab		30
	*Patella caerulea*	16	20	CR (Ma)		10
	*Hexaplex trunculus*	13	50	Ve, Ma		15
	*Galeodea echinophora*	9	10	Ab, Mo		5
	*Rapana venosa*	6	5	ER		5
	*Aporrhais pespelecani*	3	50	ER		30
	*Tonna galea*	2	3	Ab, Mo		3
	Mixed gastropods species	2	30	Ma, Mo		20
	Tot.	104				
Arthropods	*Callinectes sapidus*	30	10	FVG, Ve, ER, Ma	January 2023– March 2025	5
	*Brachyura* spp.	1	3	ER		3
	*Maja squinado*	1	2	ER		2
	*Squilla mantis*	1	1	ER		1
	Tot.	33				
Echinoderms	*Paracentrotus lividus*	23	10	CR (Ma)	May–July 2023–2024	10
	Tot.	23				

* FVG = Friuli–Venezia Giulia region, Ve = Veneto region, ER = Emila-Romagna region, Ma = Marche region, Ab = Abruzzo region, Mo = Molise region, CR = Conero Riviera.

**Table 2:** Results obtained by the IZSUM’s laboratory from participation in the international proficiency test distributed by WEPAL-QUASIMEME.

Element	Unit	Sample ID	Sample Type	Reported Value (µg kg^−1^)	NDA Mean (µg kg^−1^)	NDA Sd	N° Obs	Total Error (µg kg^−1^)	Z’-Score
TTXs	µg L^−1^	QTT012SS	Standard solution	81.85	76.6	4.4	12	9.8	0.5
	µg kg^−1^	QTT014BT	Mollusk homogenate	225.4	254	81	13	44	−0.7
	µg kg^−1^	QTT013BT	Mollusk homogenate	n.d. *	n.d. *	/	/	/	/

* ID = identifier, NDA Mean = Normal Distribution Approximation Mean, NDA Sd = Normal Distribution Approximation standard deviation, N° Obs = number of observations, n.d. = not detectable.

**Table 3:** Calibration curves, matrix effect, and matrix equivalence of the optimized HILIC-MS/MS method.

Matrix	Curve	R^2^	ME (%) *	EQM (%) **	t_calc_ ***	t_table_ ***
Solvent	y = 593.07x + 675.93	0.9949	/	/	/	
Mussels	y = 180.20x − 35.85	0.9942	30	/	/	
Gastropods	y = 178.74x + 48.41	0.9944	30	99	0.41	2.10
Echinoderms	y = 174.35x − 41.28	0.9986	29	97	1.87	
Arthropods	y = 171.17x − 139.30	0.9990	29	95	1.92	

* ME% = slope of matrix-matched standard curve/slope of solvent standard curve × 100. ** EQM% = slope of matrix-matched standard curve/slope of mussel-matched standard × 100. *** t_calc_.= calculated t-value, t_table_ = table t-value (*p* < 0.05).

## Data Availability

The original contributions presented in the study are included in the article/Supplementary Materials. Further inquiries can be directed to the corresponding author.

## Supplementary Materials

The following supporting information can be downloaded at https://www.mdpi.com/article/10.3390/foods14234036/s1, Table S1: Samples collected in North–Central Adriatic Sea between January 2023 and March 2025: results obtained by HILIC-MS/MS for all TTX analogues included in the method; Figure S1: Species under study: *Bolinus brandaris* (1), *Nassarius mutabilis* (2), *Patella caerulea* (3), *Hexaplex trunculus* (4), *Galeodea echinophora* (5), *Rapana venosa* (6), *Aporrhais pespelecani* (7), *Tonna galea* (8), *Callinectes sapidus* (9), *Brachyura* spp. (10), *Maja squinado* (11), *Squilla mantis* (12), and *Paracentrotus lividus* (13); Figure S2: A schematic diagram of the two EURLMB official protocols: the “EURLMB SOP for the analysis of Paralytic Shellfish Toxins (PST) by precolumn HPLC-FLD according to OMA AOAC 2005.06” [40] and the EURLMB SOP “Determination of Tetrodotoxin by HILIC-MS/MS” [41]; Table S2: HILIC-MS/MS method for TTXs analysis: LC, MS parameters, and transitions in multiple reaction monitoring (MRM); Figure S3: HILIC-MS/MS chromatogram of a matrix-matched standard solution (2 µg mL^−1^) containing TTX, 4,9-anhydro TTX, and non-certified analogue of 4-epi TTX. Multiple reaction monitoring (MRM) transitions were shown for each analyte; Figure S4: Mean calibration curves (replicates injected in three different days) in solvent and in the four matrices (mussels, echinoderms, gastropods, and arthropods); Table S3: Matrix equivalence of 4,9-anhydro TTX, expressed as the percentage area ratios of echinoderms, gastropods, and blue crabs relative to mussels, evaluated at two different concentration levels; Table S4: HILIC-MS/MS method performances obtained for TTX in the studied matrices at two spiking levels: concentrations (µg kg^−1^), recovery (R%), mean concentration (µg kg^−1^), and mean recovery (R%) with the corresponding standard deviation (SD), and the precision expressed as inter-day relative standard deviation (RSD_R_%).
