# Supplementary material for: Survey of Tetrodotoxins (TTXs) in Gastropods, Sea Urchins, and Blue Crabs from the Adriatic Sea: First Report in Paracentrotus lividus
Source: Foods. 2025 Nov 25;14(23):4036. doi: 10.3390/foods14234036 (PMC12691885; doi:10.3390/foods14234036)
Supplement: Supplementary file 1 [file foods-14-04036-s001.zip › foods-3972006-supplementary.pdf]

Table S1. Samples collected in North-Central Adriatic Sea between January 2023 and March 2025: results obtained by HILIC-MS/MS for all TTX analogues included in the method.

Date	Species	Sampling area*	TTX µg kg-1	4-epi TTX µg kg-1	11-nor TTX-6-ol µg kg-1	6,11-dideoxyTTX µg kg-1	5-Deoxy TTX µg kg-1	11-Deoxy TTX µg kg-1	11-oxo-TTX µg kg-1	5,6,11 Trideoxy TTX µg kg-1	4,9-anhydro TTX µg kg-1
06/01/2023	*H. trunculus*	Ve	N.D (<3)	N.D (<3)	N.D (<3)	N.D (<3)	N.D (<3)	N.D (<3)	N.D (<3)	N.D (<3)	N.D (<3)
28/02/2023	*B. brandaris*	ER	N.D (<3)	N.D (<3)	N.D (<3)	N.D (<3)	N.D (<3)	N.D (<3)	N.D (<3)	N.D (<3)	N.D (<3)
21/03/2023	*B. brandaris*	Ma	N.D (<3)	N.D (<3)	N.D (<3)	N.D (<3)	N.D (<3)	N.D (<3)	N.D (<3)	N.D (<3)	N.D (<3)
21/03/2023	*B. brandaris*	Ma	N.D (<3)	N.D (<3)	N.D (<3)	N.D (<3)	N.D (<3)	N.D (<3)	N.D (<3)	N.D (<3)	N.D (<3)
21/03/2023	*N. mutabilis*	ER	N.D (<3)	N.D (<3)	N.D (<3)	N.D (<3)	N.D (<3)	N.D (<3)	N.D (<3)	N.D (<3)	N.D (<3)
21/03/2023	*N. mutabilis*	ER	N.D (<3)	N.D (<3)	N.D (<3)	N.D (<3)	N.D (<3)	N.D (<3)	N.D (<3)	N.D (<3)	N.D (<3)
23/03/2023	*N. mutabilis*	ER	N.D (<3)	N.D (<3)	N.D (<3)	N.D (<3)	N.D (<3)	N.D (<3)	N.D (<3)	N.D (<3)	N.D (<3)
24/03/2023	*N. mutabilis*	ER	N.D (<3)	N.D (<3)	N.D (<3)	N.D (<3)	N.D (<3)	N.D (<3)	N.D (<3)	N.D (<3)	N.D (<3)
24/03/2023	*N. mutabilis*	ER	N.D (<3)	N.D (<3)	N.D (<3)	N.D (<3)	N.D (<3)	N.D (<3)	N.D (<3)	N.D (<3)	N.D (<3)
24/03/2023	*N. mutabilis*	ER	N.D (<3)	N.D (<3)	N.D (<3)	N.D (<3)	N.D (<3)	N.D (<3)	N.D (<3)	N.D (<3)	N.D (<3)
01/05/2023	*G.echinophora*	Mo	N.D (<3)	N.D (<3)	N.D (<3)	N.D (<3)	N.D (<3)	N.D (<3)	N.D (<3)	N.D (<3)	N.D (<3)
10/05/2023	*N. mutabilis*	Ma	N.D (<3)	N.D (<3)	N.D (<3)	N.D (<3)	N.D (<3)	N.D (<3)	N.D (<3)	N.D (<3)	N.D (<3)
12/05/2023	*P. lividus*	CR (Ma)	N.D (<3)	N.D (<3)	N.D (<3)	N.D (<3)	N.D (<3)	N.D (<3)	N.D (<3)	N.D (<3)	N.D (<3)
12/05/2023	*P. lividus*	CR (Ma)	N.D (<3)	N.D (<3)	N.D (<3)	N.D (<3)	N.D (<3)	N.D (<3)	N.D (<3)	N.D (<3)	N.D (<3)
12/05/2023	*P. lividus*	CR (Ma)	N.D (<3)	N.D (<3)	N.D (<3)	N.D (<3)	N.D (<3)	N.D (<3)	N.D (<3)	N.D (<3)	N.D (<3)
18/05/2023	*H. trunculus*	Ve	N.D (<3)	N.D (<3)	N.D (<3)	N.D (<3)	N.D (<3)	N.D (<3)	N.D (<3)	N.D (<3)	N.D (<3)
23/05/2023	*N. mutabilis*	Ab	N.D (<3)	N.D (<3)	N.D (<3)	N.D (<3)	N.D (<3)	N.D (<3)	N.D (<3)	N.D (<3)	N.D (<3)
24/05/2023	*B. brandaris*	ER	N.D (<3)	N.D (<3)	N.D (<3)	N.D (<3)	N.D (<3)	N.D (<3)	N.D (<3)	N.D (<3)	N.D (<3)
29/05/2023	*P. caerulea*	CR (Ma)	N.D (<3)	N.D (<3)	N.D (<3)	N.D (<3)	N.D (<3)	N.D (<3)	N.D (<3)	N.D (<3)	N.D (<3)
29/05/2023	*P. caerulea*	CR (Ma)	N.D (<3)	N.D (<3)	N.D (<3)	N.D (<3)	N.D (<3)	N.D (<3)	N.D (<3)	N.D (<3)	N.D (<3)
29/05/2023	Mixed gastropods species	Ma	N.D (<3)	N.D (<3)	N.D (<3)	N.D (<3)	N.D (<3)	N.D (<3)	N.D (<3)	N.D (<3)	N.D (<3)
29/05/2023	*P. lividus*	CR (Ma)	N.D (<3)	N.D (<3)	N.D (<3)	N.D (<3)	N.D (<3)	N.D (<3)	N.D (<3)	N.D (<3)	N.D (<3)
01/06/2023	Mixed gastropods species	Mo	N.D (<3)	N.D (<3)	N.D (<3)	N.D (<3)	N.D (<3)	N.D (<3)	N.D (<3)	N.D (<3)	N.D (<3)
07/06/2023	*N. mutabilis*	ER	N.D (<3)	N.D (<3)	N.D (<3)	N.D (<3)	N.D (<3)	N.D (<3)	N.D (<3)	N.D (<3)	N.D (<3)
08/06/2023	*R. venosa*	ER	N.D (<3)	N.D (<3)	N.D (<3)	N.D (<3)	N.D (<3)	N.D (<3)	N.D (<3)	N.D (<3)	N.D (<3)
08/06/2023	*B. brandaris*	ER	N.D (<3)	N.D (<3)	N.D (<3)	N.D (<3)	N.D (<3)	N.D (<3)	N.D (<3)	N.D (<3)	N.D (<3)

Date	Species	Sampling area*	TTX µg kg^{-1}	4-epi TTX µg kg^{-1}	11-nor TTX-6-ol µg kg^{-1}	6,11-dideoxyTTX µg kg^{-1}	5-Deoxy TTX µg kg^{-1}	11-Deoxy TTX µg kg^{-1}	11-oxo-TTX µg kg^{-1}	5,6,11 Trideoxy TTX µg kg^{-1}	4,9-anhydro TTX µg kg^{-1}
08/06/2023	*B. brandaris*	Ma	N.D (<3)	N.D (<3)	N.D (<3)	N.D (<3)	N.D (<3)	N.D (<3)	N.D (<3)	N.D (<3)	N.D (<3)
13/06/2023	*P. caerulea*	CR (Ma)	N.D (<3)	N.D (<3)	N.D (<3)	N.D (<3)	N.D (<3)	N.D (<3)	N.D (<3)	N.D (<3)	N.D (<3)
13/06/2023	*N. mutabilis*	Ma	N.D (<3)	N.D (<3)	N.D (<3)	N.D (<3)	N.D (<3)	N.D (<3)	N.D (<3)	N.D (<3)	N.D (<3)
13/06/2023	*P. caerulea*	CR (Ma)	N.D (<3)	N.D (<3)	N.D (<3)	N.D (<3)	N.D (<3)	N.D (<3)	N.D (<3)	N.D (<3)	N.D (<3)
13/06/2023	*P. lividus*	CR (Ma)	N.D (<3)	N.D (<3)	N.D (<3)	N.D (<3)	N.D (<3)	N.D (<3)	N.D (<3)	N.D (<3)	N.D (<3)
14/06/2023	*N. mutabilis*	ER	N.D (<3)	N.D (<3)	N.D (<3)	N.D (<3)	N.D (<3)	N.D (<3)	N.D (<3)	N.D (<3)	N.D (<3)
19/06/2023	*P. caerulea*	CR (Ma)	N.D (<3)	N.D (<3)	N.D (<3)	N.D (<3)	N.D (<3)	N.D (<3)	N.D (<3)	N.D (<3)	N.D (<3)
19/06/2023	*P. caerulea*	CR (Ma)	N.D (<3)	N.D (<3)	N.D (<3)	N.D (<3)	N.D (<3)	N.D (<3)	N.D (<3)	N.D (<3)	N.D (<3)
19/06/2023	*P. lividus*	CR (Ma)	N.D (<3)	N.D (<3)	N.D (<3)	N.D (<3)	N.D (<3)	N.D (<3)	N.D (<3)	N.D (<3)	N.D (<3)
19/06/2023	*P. lividus*	CR (Ma)	N.D (<3)	N.D (<3)	N.D (<3)	N.D (<3)	N.D (<3)	N.D (<3)	N.D (<3)	N.D (<3)	N.D (<3)
20/06/2023	*B. brandaris*	ER	N.D (<3)	N.D (<3)	N.D (<3)	N.D (<3)	N.D (<3)	N.D (<3)	N.D (<3)	N.D (<3)	N.D (<3)
21/06/2023	*H. trunculus*	Ve	N.D (<3)	N.D (<3)	N.D (<3)	N.D (<3)	N.D (<3)	N.D (<3)	N.D (<3)	N.D (<3)	N.D (<3)
21/06/2023	*H. trunculus*	Ve	N.D (<3)	N.D (<3)	N.D (<3)	N.D (<3)	N.D (<3)	N.D (<3)	N.D (<3)	N.D (<3)	N.D (<3)
27/06/2023	*P. caerulea*	CR (Ma)	N.D (<3)	N.D (<3)	N.D (<3)	N.D (<3)	N.D (<3)	N.D (<3)	N.D (<3)	N.D (<3)	N.D (<3)
27/06/2023	*P. caerulea*	CR (Ma)	N.D (<3)	N.D (<3)	N.D (<3)	N.D (<3)	N.D (<3)	N.D (<3)	N.D (<3)	N.D (<3)	N.D (<3)
27/06/2023	*P. caerulea*	CR (Ma)	N.D (<3)	N.D (<3)	N.D (<3)	N.D (<3)	N.D (<3)	N.D (<3)	N.D (<3)	N.D (<3)	N.D (<3)
27/06/2023	*P. caerulea*	CR (Ma)	N.D (<3)	N.D (<3)	N.D (<3)	N.D (<3)	N.D (<3)	N.D (<3)	N.D (<3)	N.D (<3)	N.D (<3)
27/06/2023	*P. lividus*	CR (Ma)	N.D (<3)	N.D (<3)	N.D (<3)	N.D (<3)	N.D (<3)	N.D (<3)	N.D (<3)	N.D (<3)	N.D (<3)
01/07/2023	*T. galea*	Mo	N.D (<3)	N.D (<3)	N.D (<3)	N.D (<3)	N.D (<3)	N.D (<3)	N.D (<3)	N.D (<3)	N.D (<3)
04/07/2023	*T. galea*	Ab	N.D (<3)	N.D (<3)	N.D (<3)	N.D (<3)	N.D (<3)	N.D (<3)	N.D (<3)	N.D (<3)	N.D (<3)
06/07/2023	*P. caerulea*	CR (Ma)	N.D (<3)	N.D (<3)	N.D (<3)	N.D (<3)	N.D (<3)	N.D (<3)	N.D (<3)	N.D (<3)	N.D (<3)
06/07/2023	*P. caerulea*	CR (Ma)	N.D (<3)	N.D (<3)	N.D (<3)	N.D (<3)	N.D (<3)	N.D (<3)	N.D (<3)	N.D (<3)	N.D (<3)
06/07/2023	*P. caerulea*	CR (Ma)	N.D (<3)	N.D (<3)	N.D (<3)	N.D (<3)	N.D (<3)	N.D (<3)	N.D (<3)	N.D (<3)	N.D (<3)
06/07/2023	*P. caerulea*	CR (Ma)	N.D (<3)	N.D (<3)	N.D (<3)	N.D (<3)	N.D (<3)	N.D (<3)	N.D (<3)	N.D (<3)	N.D (<3)
06/07/2023	*P. lividus*	CR (Ma)	N.D (<3)	N.D (<3)	N.D (<3)	N.D (<3)	N.D (<3)	N.D (<3)	N.D (<3)	N.D (<3)	N.D (<3)
07/07/2023	*B. brandaris*	Ab	N.D (<3)	N.D (<3)	N.D (<3)	N.D (<3)	N.D (<3)	N.D (<3)	N.D (<3)	N.D (<3)	N.D (<3)
12/07/2023	*P. caerulea*	CR (Ma)	N.D (<3)	N.D (<3)	N.D (<3)	N.D (<3)	N.D (<3)	N.D (<3)	N.D (<3)	N.D (<3)	N.D (<3)
12/07/2023	*P. caerulea*	CR (Ma)	N.D (<3)	N.D (<3)	N.D (<3)	N.D (<3)	N.D (<3)	N.D (<3)	N.D (<3)	N.D (<3)	N.D (<3)
12/07/2023	*B. brandaris*	ER	N.D (<3)	N.D (<3)	N.D (<3)	N.D (<3)	N.D (<3)	N.D (<3)	N.D (<3)	N.D (<3)	N.D (<3)
12/07/2023	*R. venosa*	ER	N.D (<3)	N.D (<3)	N.D (<3)	N.D (<3)	N.D (<3)	N.D (<3)	N.D (<3)	N.D (<3)	N.D (<3)
12/07/2023	*C. sapidus*	ER	N.D (<5)	N.D (<5)	N.D (<5)	N.D (<5)	N.D (<5)	N.D (<5)	N.D (<5)	N.D (<5)	N.D (<5)
12/07/2023	*P. lividus*	CR (Ma)	**~5**	N.D (<3)	N.D (<3)	N.D (<3)	N.D (<3)	N.D (<3)	N.D (<3)	N.D (<3)	N.D (<3)

Date	Species	Sampling area*	TTX µg kg^{-1}	4-epi TTX µg kg^{-1}	11-nor TTX-6-ol µg kg^{-1}	6,11-dideoxyTTX µg kg^{-1}	5-Deoxy TTX µg kg^{-1}	11-Deoxy TTX µg kg^{-1}	11-oxo-TTX µg kg^{-1}	5,6,11 Trideoxy TTX µg kg^{-1}	4,9-anhydro TTX µg kg^{-1}
20/07/2023	*C. sapidus*	ER	N.D (<5)	N.D (<5)	N.D (<5)	N.D (<5)	N.D (<5)	N.D (<5)	N.D (<5)	N.D (<5)	N.D (<5)
29/07/2023	*B. brandaris*	Ab	N.D (<3)	N.D (<3)	N.D (<3)	N.D (<3)	N.D (<3)	N.D (<3)	N.D (<3)	N.D (<3)	N.D (<3)
01/08/2023	*G.echinophora*	Ab	N.D (<3)	N.D (<3)	N.D (<3)	N.D (<3)	N.D (<3)	N.D (<3)	N.D (<3)	N.D (<3)	N.D (<3)
11/08/2023	*G.echinophora*	Ab	N.D (<3)	N.D (<3)	N.D (<3)	N.D (<3)	N.D (<3)	N.D (<3)	N.D (<3)	N.D (<3)	N.D (<3)
01/09/2023	*G.echinophora*	Ab	N.D (<3)	N.D (<3)	N.D (<3)	N.D (<3)	N.D (<3)	N.D (<3)	N.D (<3)	N.D (<3)	N.D (<3)
22/09/2023	*B. brandaris*	Ab	N.D (<3)	N.D (<3)	N.D (<3)	N.D (<3)	N.D (<3)	N.D (<3)	N.D (<3)	N.D (<3)	N.D (<3)
01/11/2023	*G.echinophora*	Ab	N.D (<3)	N.D (<3)	N.D (<3)	N.D (<3)	N.D (<3)	N.D (<3)	N.D (<3)	N.D (<3)	N.D (<3)
28/11/2023	*B. brandaris*	Ma	N.D (<3)	N.D (<3)	N.D (<3)	N.D (<3)	N.D (<3)	N.D (<3)	N.D (<3)	N.D (<3)	N.D (<3)
11/12/2023	*N. mutabilis*	ER	N.D (<3)	N.D (<3)	N.D (<3)	N.D (<3)	N.D (<3)	N.D (<3)	N.D (<3)	N.D (<3)	N.D (<3)
13/12/2023	*C. sapidus*	ER	N.D (<5)	N.D (<5)	N.D (<5)	N.D (<5)	N.D (<5)	N.D (<5)	N.D (<5)	N.D (<5)	N.D (<5)
14/12/2023	*B. brandaris*	Ma	N.D (<3)	N.D (<3)	N.D (<3)	N.D (<3)	N.D (<3)	N.D (<3)	N.D (<3)	N.D (<3)	N.D (<3)
15/12/2023	*N. mutabilis*	Ma	N.D (<3)	N.D (<3)	N.D (<3)	N.D (<3)	N.D (<3)	N.D (<3)	N.D (<3)	N.D (<3)	N.D (<3)
21/12/2023	*H. trunculus*	Ve	N.D (<3)	N.D (<3)	N.D (<3)	N.D (<3)	N.D (<3)	N.D (<3)	N.D (<3)	N.D (<3)	N.D (<3)
01/01/2024	*C. sapidus*	ER	N.D (<5)	N.D (<5)	N.D (<5)	N.D (<5)	N.D (<5)	N.D (<5)	N.D (<5)	N.D (<5)	N.D (<5)
19/01/2024	*C. sapidus*	ER	N.D (<5)	N.D (<5)	N.D (<5)	N.D (<5)	N.D (<5)	N.D (<5)	N.D (<5)	N.D (<5)	N.D (<5)
01/02/2024	*B. brandaris*	Ma	N.D (<3)	N.D (<3)	N.D (<3)	N.D (<3)	N.D (<3)	N.D (<3)	N.D (<3)	N.D (<3)	N.D (<3)
06/02/2024	*B. brandaris*	Ma	N.D (<3)	N.D (<3)	N.D (<3)	N.D (<3)	N.D (<3)	N.D (<3)	N.D (<3)	N.D (<3)	N.D (<3)
12/02/2024	*C. sapidus*	ER	N.D (<5)	N.D (<5)	N.D (<5)	N.D (<5)	N.D (<5)	N.D (<5)	N.D (<5)	N.D (<5)	N.D (<5)
19/02/2024	*N. mutabilis*	Ma	N.D (<3)	N.D (<3)	N.D (<3)	N.D (<3)	N.D (<3)	N.D (<3)	N.D (<3)	N.D (<3)	N.D (<3)
26/02/2024	*R. venosa*	ER	N.D (<3)	N.D (<3)	N.D (<3)	N.D (<3)	N.D (<3)	N.D (<3)	N.D (<3)	N.D (<3)	N.D (<3)
06/03/2024	*R. venosa*	ER	N.D (<3)	N.D (<3)	N.D (<3)	N.D (<3)	N.D (<3)	N.D (<3)	N.D (<3)	N.D (<3)	N.D (<3)
06/03/2024	*A. pespelecani*	ER	N.D (<3)	N.D (<3)	N.D (<3)	N.D (<3)	N.D (<3)	N.D (<3)	N.D (<3)	N.D (<3)	N.D (<3)
06/03/2024	*C. sapidus*	ER	N.D (<5)	N.D (<5)	N.D (<5)	N.D (<5)	N.D (<5)	N.D (<5)	N.D (<5)	N.D (<5)	N.D (<5)
06/03/2024	*C. sapidus*	ER	N.D (<5)	N.D (<5)	N.D (<5)	N.D (<5)	N.D (<5)	N.D (<5)	N.D (<5)	N.D (<5)	N.D (<5)
06/03/2024	*C. sapidus*	Ve	N.D (<5)	N.D (<5)	N.D (<5)	N.D (<5)	N.D (<5)	N.D (<5)	N.D (<5)	N.D (<5)	N.D (<5)
07/03/2024	*R. venosa*	ER	N.D (<3)	N.D (<3)	N.D (<3)	N.D (<3)	N.D (<3)	N.D (<3)	N.D (<3)	N.D (<3)	N.D (<3)
07/03/2024	*A. pespelecani*	ER	N.D (<3)	N.D (<3)	N.D (<3)	N.D (<3)	N.D (<3)	N.D (<3)	N.D (<3)	N.D (<3)	N.D (<3)
07/03/2024	*C. sapidus*	ER	N.D (<5)	N.D (<5)	N.D (<5)	N.D (<5)	N.D (<5)	N.D (<5)	N.D (<5)	N.D (<5)	N.D (<5)
11/03/2024	*C. sapidus*	ER	N.D (<5)	N.D (<5)	N.D (<5)	N.D (<5)	N.D (<5)	N.D (<5)	N.D (<5)	N.D (<5)	N.D (<5)
18/03/2024	*C. sapidus*	ER	N.D (<5)	N.D (<5)	N.D (<5)	N.D (<5)	N.D (<5)	N.D (<5)	N.D (<5)	N.D (<5)	N.D (<5)
26/03/2024	*C. sapidus*	ER	N.D (<5)	N.D (<5)	N.D (<5)	N.D (<5)	N.D (<5)	N.D (<5)	N.D (<5)	N.D (<5)	N.D (<5)
26/03/2024	*C. sapidus*	Ve	N.D (<5)	N.D (<5)	N.D (<5)	N.D (<5)	N.D (<5)	N.D (<5)	N.D (<5)	N.D (<5)	N.D (<5)

Date	Species	Sampling area*	TTX µg kg^{-1}	4-epi TTX µg kg^{-1}	11-nor TTX-6-ol µg kg^{-1}	6,11-dideoxyTTX µg kg^{-1}	5-Deoxy TTX µg kg^{-1}	11-Deoxy TTX µg kg^{-1}	11-oxo-TTX µg kg^{-1}	5,6,11 Trideoxy TTX µg kg^{-1}	4,9-anhydro TTX µg kg^{-1}
02/04/2024	*C. sapidus*	Ve	N.D (<5)	N.D (<5)	N.D (<5)	N.D (<5)	N.D (<5)	N.D (<5)	N.D (<5)	N.D (<5)	N.D (<5)
04/04/2024	*C. sapidus*	ER	N.D (<5)	N.D (<5)	N.D (<5)	N.D (<5)	N.D (<5)	N.D (<5)	N.D (<5)	N.D (<5)	N.D (<5)
08/04/2024	*C. sapidus*	FVG	N.D (<5)	N.D (<5)	N.D (<5)	N.D (<5)	N.D (<5)	N.D (<5)	N.D (<5)	N.D (<5)	N.D (<5)
10/04/2024	*C. sapidus*	ER	N.D (<5)	N.D (<5)	N.D (<5)	N.D (<5)	N.D (<5)	N.D (<5)	N.D (<5)	N.D (<5)	N.D (<5)
19/04/2024	*C. sapidus*	ER	N.D (<5)	N.D (<5)	N.D (<5)	N.D (<5)	N.D (<5)	N.D (<5)	N.D (<5)	N.D (<5)	N.D (<5)
21/04/2024	*H. trunculus*	Ve	N.D (<3)	N.D (<3)	N.D (<3)	N.D (<3)	N.D (<3)	N.D (<3)	N.D (<3)	N.D (<3)	N.D (<3)
06/05/2024	*N. mutabilis*	Ma	N.D (<3)	N.D (<3)	N.D (<3)	N.D (<3)	N.D (<3)	N.D (<3)	N.D (<3)	N.D (<3)	N.D (<3)
07/05/2024	*C. sapidus*	ER	N.D (<5)	N.D (<5)	N.D (<5)	N.D (<5)	N.D (<5)	N.D (<5)	N.D (<5)	N.D (<5)	N.D (<5)
10/05/2024	*C. sapidus*	ER	N.D (<5)	N.D (<5)	N.D (<5)	N.D (<5)	N.D (<5)	N.D (<5)	N.D (<5)	N.D (<5)	N.D (<5)
14/05/2024	*C. sapidus*	Ve	N.D (<5)	N.D (<5)	N.D (<5)	N.D (<5)	N.D (<5)	N.D (<5)	N.D (<5)	N.D (<5)	N.D (<5)
16/05/2024	*B. brandaris*	Ve	N.D (<3)	N.D (<3)	N.D (<3)	N.D (<3)	N.D (<3)	N.D (<3)	N.D (<3)	N.D (<3)	N.D (<3)
26/05/2024	*H. trunculus*	Ve	N.D (<3)	N.D (<3)	N.D (<3)	N.D (<3)	N.D (<3)	N.D (<3)	N.D (<3)	N.D (<3)	N.D (<3)
29/05/2024	*C. sapidus*	ER	N.D (<3)	N.D (<3)	N.D (<3)	N.D (<3)	N.D (<3)	N.D (<3)	N.D (<3)	N.D (<3)	N.D (<3)
31/05/2024	*P. lividus*	CR (Ma)	N.D (<3)	N.D (<3)	N.D (<3)	N.D (<3)	N.D (<3)	N.D (<3)	N.D (<3)	N.D (<3)	N.D (<3)
31/05/2024	*P. lividus*	CR (Ma)	N.D (<3)	N.D (<3)	N.D (<3)	N.D (<3)	N.D (<3)	N.D (<3)	N.D (<3)	N.D (<3)	N.D (<3)
09/06/2024	*H. trunculus*	Ve	N.D (<3)	N.D (<3)	N.D (<3)	N.D (<3)	N.D (<3)	N.D (<3)	N.D (<3)	N.D (<3)	N.D (<3)
10/06/2024	*P. lividus*	CR (Ma)	N.D (<3)	N.D (<3)	N.D (<3)	N.D (<3)	N.D (<3)	N.D (<3)	N.D (<3)	N.D (<3)	N.D (<3)
10/06/2024	*P. lividus*	CR (Ma)	N.D (<3)	N.D (<3)	N.D (<3)	N.D (<3)	N.D (<3)	N.D (<3)	N.D (<3)	N.D (<3)	N.D (<3)
10/06/2024	*P. lividus*	CR (Ma)	N.D (<3)	N.D (<3)	N.D (<3)	N.D (<3)	N.D (<3)	N.D (<3)	N.D (<3)	N.D (<3)	N.D (<3)
26/06/2024	*P. lividus*	CR (Ma)	N.D (<3)	N.D (<3)	N.D (<3)	N.D (<3)	N.D (<3)	N.D (<3)	N.D (<3)	N.D (<3)	N.D (<3)
26/06/2024	*P. lividus*	CR (Ma)	N.D (<3)	N.D (<3)	N.D (<3)	N.D (<3)	N.D (<3)	N.D (<3)	N.D (<3)	N.D (<3)	N.D (<3)
26/06/2024	*P. lividus*	CR (Ma)	N.D (<3)	N.D (<3)	N.D (<3)	N.D (<3)	N.D (<3)	N.D (<3)	N.D (<3)	N.D (<3)	N.D (<3)
01/07/2024	*P. lividus*	CR (Ma)	N.D (<3)	N.D (<3)	N.D (<3)	N.D (<3)	N.D (<3)	N.D (<3)	N.D (<3)	N.D (<3)	N.D (<3)
01/07/2024	*P. lividus*	CR (Ma)	N.D (<3)	N.D (<3)	N.D (<3)	N.D (<3)	N.D (<3)	N.D (<3)	N.D (<3)	N.D (<3)	N.D (<3)
12/07/2024	*C. sapidus*	ER	N.D (<3)	N.D (<3)	N.D (<3)	N.D (<3)	N.D (<3)	N.D (<3)	N.D (<3)	N.D (<3)	N.D (<3)
14/07/2024	*H. trunculus*	Ve	N.D (<3)	N.D (<3)	N.D (<3)	N.D (<3)	N.D (<3)	N.D (<3)	N.D (<3)	N.D (<3)	N.D (<3)
15/07/2024	*C. sapidus*	ER	N.D (<3)	N.D (<3)	N.D (<3)	N.D (<3)	N.D (<3)	N.D (<3)	N.D (<3)	N.D (<3)	N.D (<3)
17/07/2024	*B. brandaris*	CR (Ma)	N.D (<3)	N.D (<3)	N.D (<3)	N.D (<3)	N.D (<3)	N.D (<3)	N.D (<3)	N.D (<3)	N.D (<3)
17/07/2024	*B. brandaris*	CR (Ma)	N.D (<3)	N.D (<3)	N.D (<3)	N.D (<3)	N.D (<3)	N.D (<3)	N.D (<3)	N.D (<3)	N.D (<3)
17/07/2024	*C. sapidus*	ER	N.D (<3)	N.D (<3)	N.D (<3)	N.D (<3)	N.D (<3)	N.D (<3)	N.D (<3)	N.D (<3)	N.D (<3)
17/07/2024	*P. lividus*	CR (Ma)	N.D (<3)	N.D (<3)	N.D (<3)	N.D (<3)	N.D (<3)	N.D (<3)	N.D (<3)	N.D (<3)	N.D (<3)
17/07/2024	*P. lividus*	CR (Ma)	N.D (<3)	N.D (<3)	N.D (<3)	N.D (<3)	N.D (<3)	N.D (<3)	N.D (<3)	N.D (<3)	N.D (<3)

Date	Species	Sampling area*	TTX µg kg^{-1}	4-epi TTX µg kg^{-1}	11-nor TTX-6-ol µg kg^{-1}	6,11-dideoxyTTX µg kg^{-1}	5-Deoxy TTX µg kg^{-1}	11-Deoxy TTX µg kg^{-1}	11-oxo-TTX µg kg^{-1}	5,6,11 Trideoxy TTX µg kg^{-1}	4,9-anhydro TTX µg kg^{-1}
17/07/2024	*P. lividus*	CR (Ma)	N.D (<3)	N.D (<3)	N.D (<3)	N.D (<3)	N.D (<3)	N.D (<3)	N.D (<3)	N.D (<3)	N.D (<3)
19/07/2024	*B. brandaris*	Ma	N.D (<3)	N.D (<3)	N.D (<3)	N.D (<3)	N.D (<3)	N.D (<3)	N.D (<3)	N.D (<3)	N.D (<3)
11/08/2024	*H. trunculus*	Ve	N.D (<3)	N.D (<3)	N.D (<3)	N.D (<3)	N.D (<3)	N.D (<3)	N.D (<3)	N.D (<3)	N.D (<3)
08/09/2024	*H. trunculus*	Ve	N.D (<3)	N.D (<3)	N.D (<3)	N.D (<3)	N.D (<3)	N.D (<3)	N.D (<3)	N.D (<3)	N.D (<3)
24/09/2024	*B. brandaris*	Ma	N.D (<3)	N.D (<3)	N.D (<3)	N.D (<3)	N.D (<3)	N.D (<3)	N.D (<3)	N.D (<3)	N.D (<3)
30/09/2024	*C. sapidus*	ER	N.D (<3)	N.D (<3)	N.D (<3)	N.D (<3)	N.D (<3)	N.D (<3)	N.D (<3)	N.D (<3)	N.D (<3)
01/10/2024	*B. brandaris*	ER	N.D (<3)	N.D (<3)	N.D (<3)	N.D (<3)	N.D (<3)	N.D (<3)	N.D (<3)	N.D (<3)	N.D (<3)
06/10/2024	*H. trunculus*	Ve	N.D (<3)	N.D (<3)	N.D (<3)	N.D (<3)	N.D (<3)	N.D (<3)	N.D (<3)	N.D (<3)	N.D (<3)
07/10/2024	*C. sapidus*	ER	N.D (<3)	N.D (<3)	N.D (<3)	N.D (<3)	N.D (<3)	N.D (<3)	N.D (<3)	N.D (<3)	N.D (<3)
22/10/2024	*B. brandaris*	Ma	N.D (<3)	N.D (<3)	N.D (<3)	N.D (<3)	N.D (<3)	N.D (<3)	N.D (<3)	N.D (<3)	N.D (<3)
30/10/2024	*G.echinophora*	Ab	N.D (<3)	N.D (<3)	N.D (<3)	N.D (<3)	N.D (<3)	N.D (<3)	N.D (<3)	N.D (<3)	N.D (<3)
07/11/2024	*B. brandaris*	Ma	N.D (<3)	N.D (<3)	N.D (<3)	N.D (<3)	N.D (<3)	N.D (<3)	N.D (<3)	N.D (<3)	N.D (<3)
07/11/2024	*H. trunculus*	Ma	N.D (<3)	N.D (<3)	N.D (<3)	N.D (<3)	N.D (<3)	N.D (<3)	N.D (<3)	N.D (<3)	N.D (<3)
07/11/2024	*C. sapidus*	Ma	N.D (<3)	N.D (<3)	N.D (<3)	N.D (<3)	N.D (<3)	N.D (<3)	N.D (<3)	N.D (<3)	N.D (<3)
13/11/2024	*B. brandaris*	Ma	N.D (<3)	N.D (<3)	N.D (<3)	N.D (<3)	N.D (<3)	N.D (<3)	N.D (<3)	N.D (<3)	N.D (<3)
13/12/2024	*G.echinophora*	Ab	N.D (<3)	N.D (<3)	N.D (<3)	N.D (<3)	N.D (<3)	N.D (<3)	N.D (<3)	N.D (<3)	N.D (<3)
18/12/2024	*N. mutabilis*	Ma	N.D (<3)	N.D (<3)	N.D (<3)	N.D (<3)	N.D (<3)	N.D (<3)	N.D (<3)	N.D (<3)	N.D (<3)
28/01/2025	*G.echinophora*	Ab	N.D (<3)	N.D (<3)	N.D (<3)	N.D (<3)	N.D (<3)	N.D (<3)	N.D (<3)	N.D (<3)	N.D (<3)
03/02/2025	*B. brandaris*	Ma	N.D (<3)	N.D (<3)	N.D (<3)	N.D (<3)	N.D (<3)	N.D (<3)	N.D (<3)	N.D (<3)	N.D (<3)
05/02/2025	*N. mutabilis*	Ab	N.D (<3)	N.D (<3)	N.D (<3)	N.D (<3)	N.D (<3)	N.D (<3)	N.D (<3)	N.D (<3)	N.D (<3)
17/02/2025	*B. brandaris*	Ma	N.D (<3)	N.D (<3)	N.D (<3)	N.D (<3)	N.D (<3)	N.D (<3)	N.D (<3)	N.D (<3)	N.D (<3)
21/02/2025	*G.echinophora*	Ab	N.D (<3)	N.D (<3)	N.D (<3)	N.D (<3)	N.D (<3)	N.D (<3)	N.D (<3)	N.D (<3)	N.D (<3)
25/02/2025	*B. brandaris*	Ma	N.D (<3)	N.D (<3)	N.D (<3)	N.D (<3)	N.D (<3)	N.D (<3)	N.D (<3)	N.D (<3)	N.D (<3)
05/03/2025	*B. brandaris*	Ma	N.D (<3)	N.D (<3)	N.D (<3)	N.D (<3)	N.D (<3)	N.D (<3)	N.D (<3)	N.D (<3)	N.D (<3)
06/03/2025	*A. pespelecani*	ER	N.D (<3)	N.D (<3)	N.D (<3)	N.D (<3)	N.D (<3)	N.D (<3)	N.D (<3)	N.D (<3)	N.D (<3)
06/03/2025	*B. brandaris*	ER	N.D (<3)	N.D (<3)	N.D (<3)	N.D (<3)	N.D (<3)	N.D (<3)	N.D (<3)	N.D (<3)	N.D (<3)
06/03/2025	*R. venosa*	ER	N.D (<3)	N.D (<3)	N.D (<3)	N.D (<3)	N.D (<3)	N.D (<3)	N.D (<3)	N.D (<3)	N.D (<3)
06/03/2025	*C. sapidus*	ER	N.D (<5)	N.D (<5)	N.D (<5)	N.D (<5)	N.D (<5)	N.D (<5)	N.D (<5)	N.D (<5)	N.D (<5)
06/03/2025	*S. mantis*	ER	N.D (<5)	N.D (<5)	N.D (<5)	N.D (<5)	N.D (<5)	N.D (<5)	N.D (<5)	N.D (<5)	N.D (<5)
06/03/2025	*M. squinado*	ER	N.D (<5)	N.D (<5)	N.D (<5)	N.D (<5)	N.D (<5)	N.D (<5)	N.D (<5)	N.D (<5)	N.D (<5)
06/03/2025	*Brachyura SPP.*	ER	N.D (<5)	N.D (<5)	N.D (<5)	N.D (<5)	N.D (<5)	N.D (<5)	N.D (<5)	N.D (<5)	N.D (<5)
07/03/2025	*N. mutabilis*	Ab	N.D (<3)	N.D (<3)	N.D (<3)	N.D (<3)	N.D (<3)	N.D (<3)	N.D (<3)	N.D (<3)	N.D (<3)

Date	Species	Sampling area*	TTX µg kg^{-1}	4-epi TTX µg kg^{-1}	11-nor TTX-6-ol µg kg^{-1}	6,11-dideoxyTTX µg kg^{-1}	5-Deoxy TTX µg kg^{-1}	11-Deoxy TTX µg kg^{-1}	11-oxo-TTX µg kg^{-1}	5,6,11 Trideoxy TTX µg kg^{-1}	4,9-anhydro TTX µg kg^{-1}
09/03/2025	*B. brandaris*	Ma	N.D (<3)	N.D (<3)	N.D (<3)	N.D (<3)	N.D (<3)	N.D (<3)	N.D (<3)	N.D (<3)	N.D (<3)
17/03/2025	*N. mutabilis*	Ma	N.D (<3)	N.D (<3)	N.D (<3)	N.D (<3)	N.D (<3)	N.D (<3)	N.D (<3)	N.D (<3)	N.D (<3)
18/03/2025	*N. mutabilis*	Ma	N.D (<3)	N.D (<3)	N.D (<3)	N.D (<3)	N.D (<3)	N.D (<3)	N.D (<3)	N.D (<3)	N.D (<3)
20/03/2025	*N. mutabilis*	ER	N.D (<3)	N.D (<3)	N.D (<3)	N.D (<3)	N.D (<3)	N.D (<3)	N.D (<3)	N.D (<3)	N.D (<3)
20/03/2025	*B. brandaris*	ER	N.D (<3)	N.D (<3)	N.D (<3)	N.D (<3)	N.D (<3)	N.D (<3)	N.D (<3)	N.D (<3)	N.D (<3)
25/03/2025	*B. brandaris*	Ma	N.D (<3)	N.D (<3)	N.D (<3)	N.D (<3)	N.D (<3)	N.D (<3)	N.D (<3)	N.D (<3)	N.D (<3)

N.D = not detected. * FVG=Friuli-Venezia Giulia region, Ve=Veneto region, ER=Emila Romagna region, Ma=Marche region, Ab=Abruzzo region, Mo=Molise region, CR= Conero Riviera.

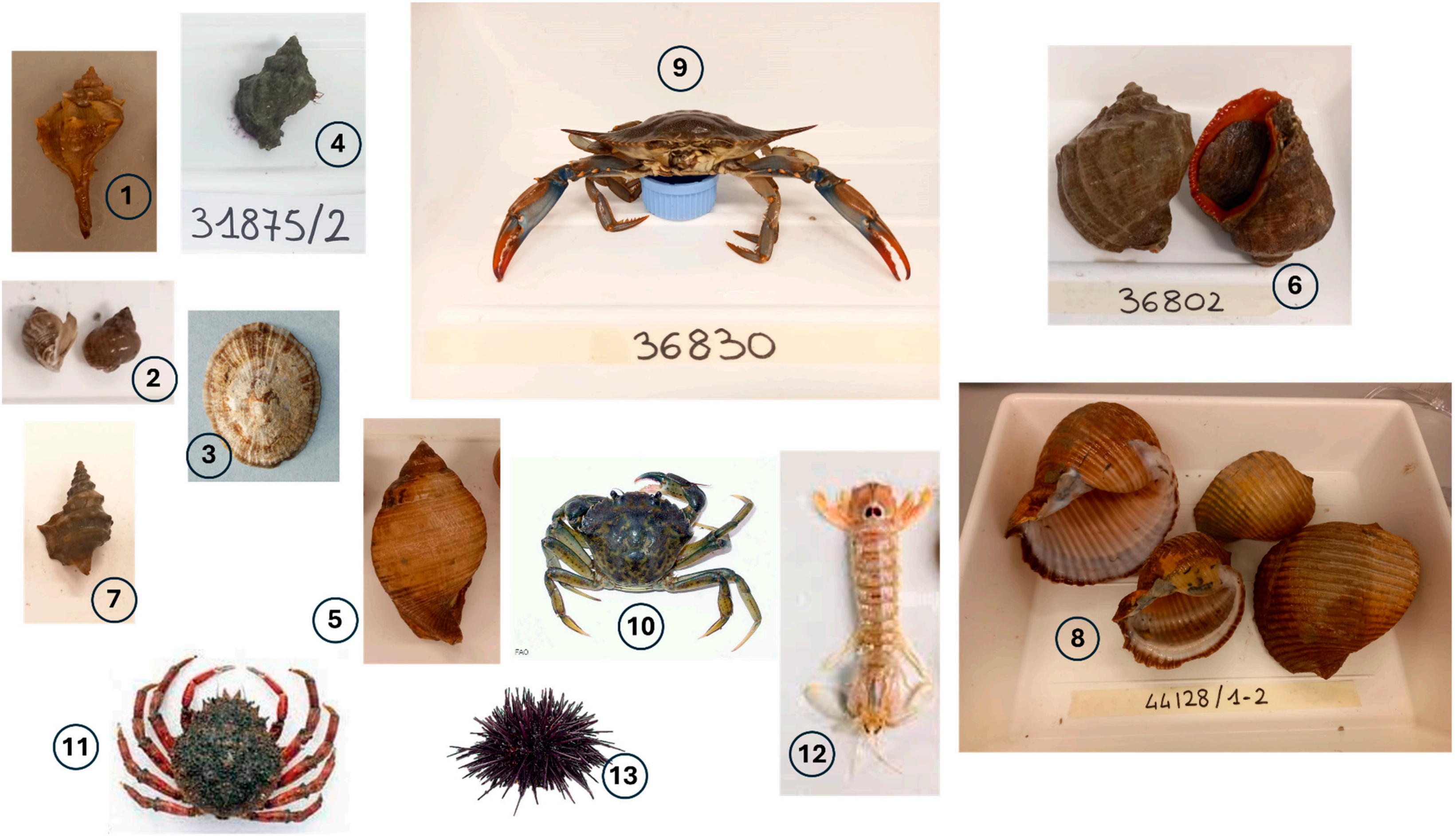

Figure S1: Species under study: *Bolinus brandaris* (1), *Nassarius mutabilis* (2), *Patella caerulea* (3), *Hexaplex trunculus* (4), *Galeodea echinophora* (5), *Rapana venosa* (6), *Aporrhais pespelecani* (7), *Tonna galea* (8), *Callinectes sapidus* (9), *Brachyura* spp. (10), *Maja squinado* (11), *Squilla mantis* (12) and *Paracentrotus lividus* (13).

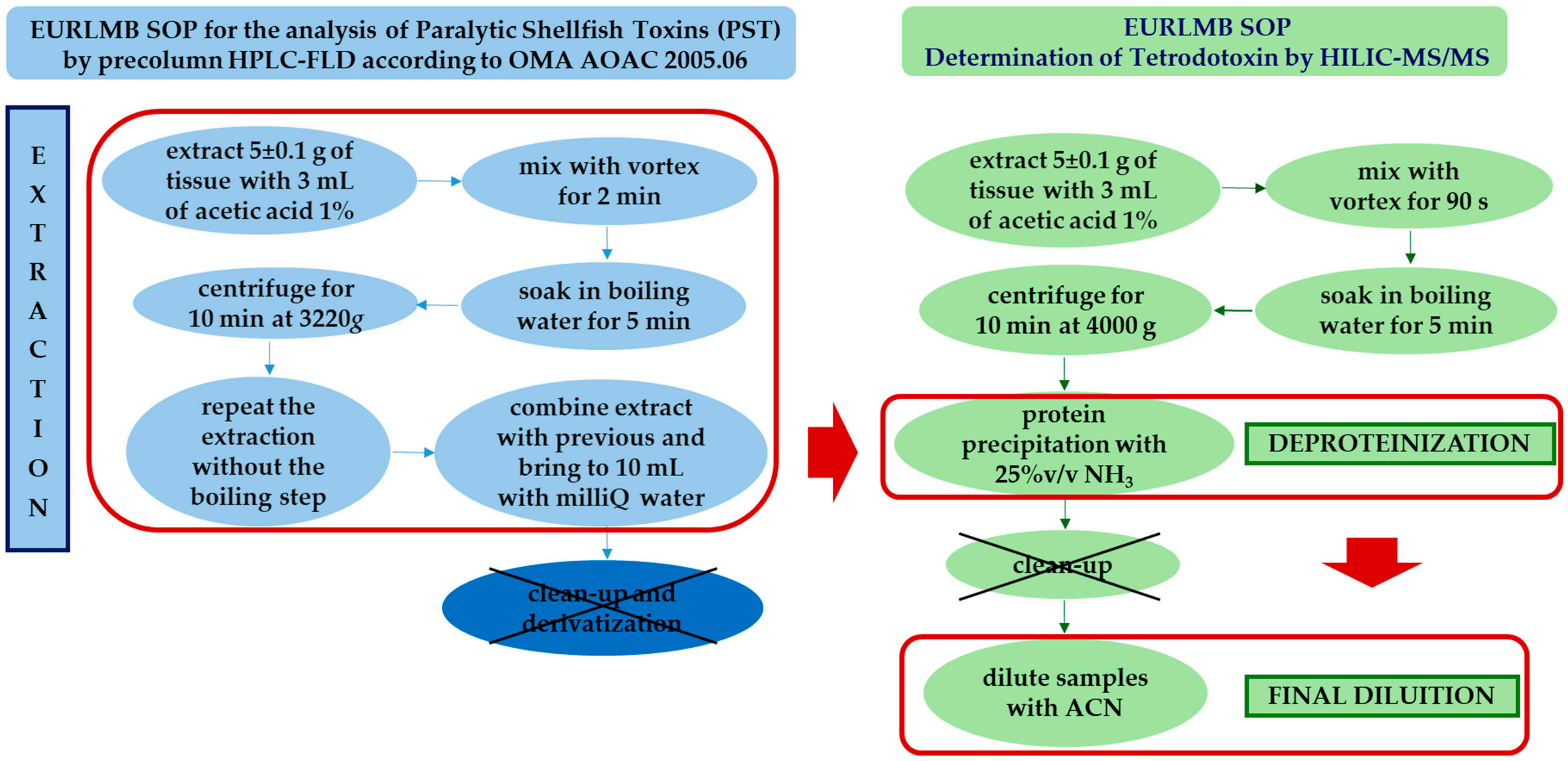

Figure S2. A schematic diagram of the two EURLMB official protocols: the "EURLMB SOP for the analysis of Paralytic Shellfish Toxins (PST) by precolumn HPLC-FLD according to OMA AOAC 2005.06" [40] and the EURLMB SOP "Determination of Tetrodotoxin by HILIC-MS/MS" [41].

Table S2. HILIC-MS/MS method for TTXs analysis: LC, MS parameters and transitions in Multiple Reaction Monitoring (MRM).

LC PARAMETERS	
**Column**	Glycan BEH Amide 130 Å 1.7µm, 2.1x150 mm (Waters)
**Injection Volume**	2 µL
**T° Column manager**	60 °C
**T° Sample manager**	6 °C
**Mobile phase A**	500 mL H_2O + 300 µL NH_4OH + 75 µL CH_2O_2
**Mobile phase B**	700 mL CH_3CN + 300 mL H_2O + 100 µL CH_2O_2

**Time (min)**	**Flow ($mLmin^{-1}$)**	**A (%)**	**B (%)**
0.00	0.4	2	98
7.00	0.4	2	98
9.50	0.4	50	50
11.00	0.5	50	50
11.50	0.5	2	98
12.00	0.6	2	98
12.50	0.6	2	98
13.00	0.4	2	98
14.00	0.4	2	98

MS/MS PARAMETERS	
**Source type**	ESI
**Capillary**	3,5 kV
**Desolvatation Temp.**	600 °C
**Desolvatation**	1000 L Hr^{-1}
**Cone**	150 L Hr^{-1}
**Source Temp.**	150 °C
**Ionization mode**	positive

MRM TRANSITIONS

**Compound**	**Prec. ion (m/z)**	**Prod. ion (m/z)**	**Dwell (sec)**	**Cone (V)**	**CE (V)**
**TTX/4-epi TTX**	320.1	302.1	0.026	40	30
		162.1[q]	0.026	40	40
**11-nor TTX-6-ol/ 6,11-dideoxy TTX**	290.1	272.1	0.026	40	30
		162.1[q]	0.026	40	30
**5-deoxy TTX/ 11-deoxy TTX**	304.1	286.1	0.026	40	30
		176.1[q]	0.026	40	30
**11-oxo-TTX**	336.1	318.1	0.026	40	30
		300.1[q]	0.026	40	30
**5,6,11-trideoxy TTX**	272.1	254.1	0.026	40	30
		162.1[q]	0.026	40	30
**4,9-anhydro TTX**	302.1	256.1	0.026	40	30
		162.1[q]	0.026	40	30

[q] MRM transition used for quantification.

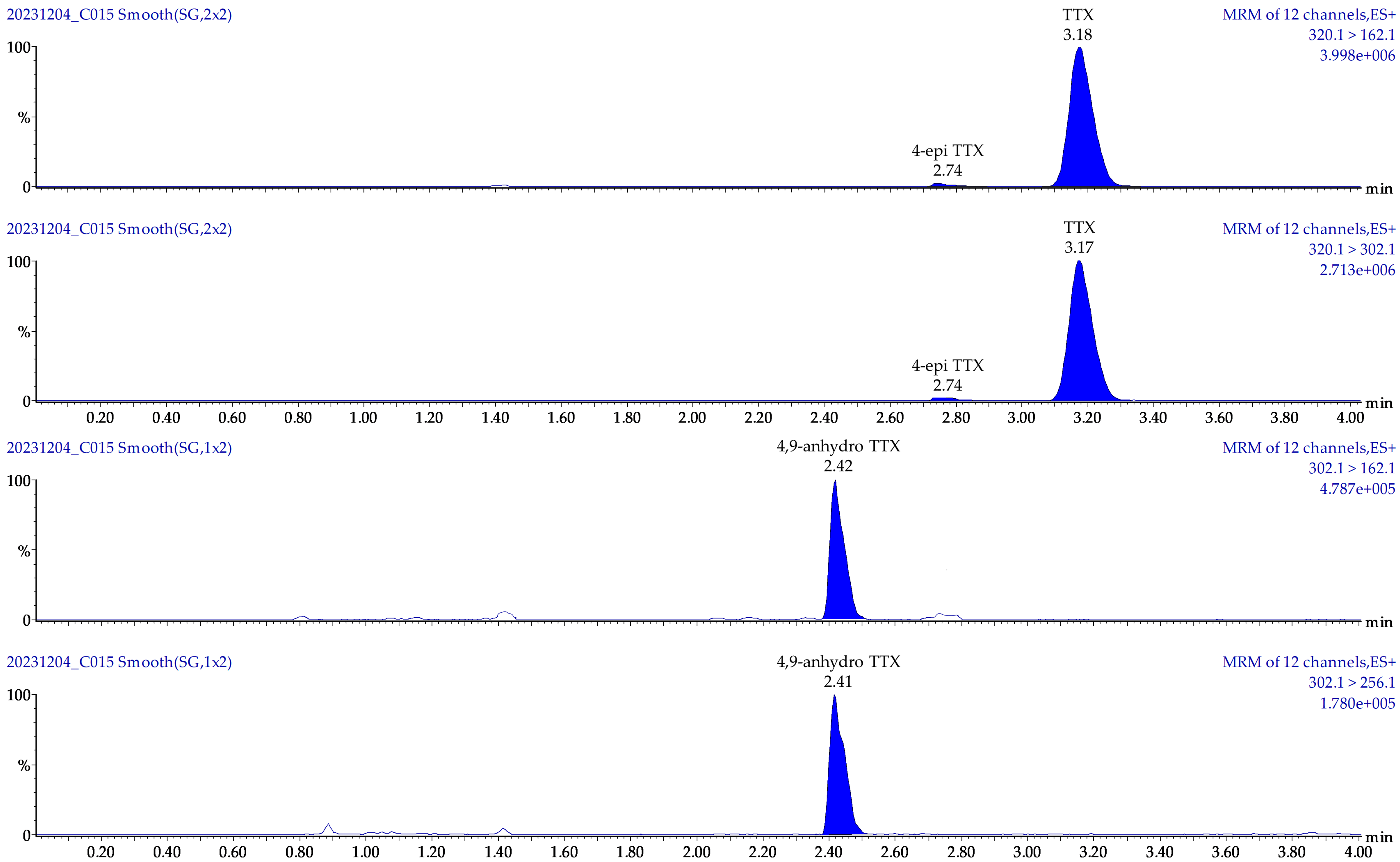

Figure S3. HILIC-MS/MS chromatogram of a matrix-matched standard solution (2 µg mL^{-1}) containing TTX, 4,9-anhydro TTX and non-certified analogue of 4-epi TTX. Multiple reaction monitoring (MRM) transitions were shown for each analyte.

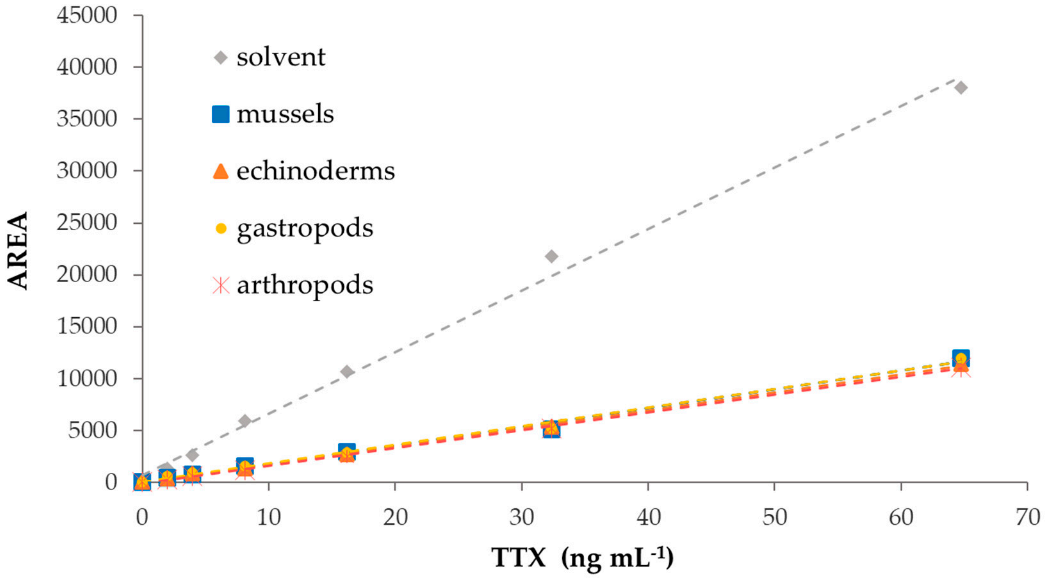

Figure S4. Mean calibration curves (replicates injected in three different days) in solvent and in the four matrices (mussels, echinoderms, gastropods, and arthropods).

Table S3. Matrix equivalence of 4,9-anhydro TTX, expressed as the percentage area ratios of echinoderms, gastropods, and blue crabs relative to mussels, evaluated at two different concentration levels.

**Matrix**	**EQM (%)** (3.73 ng mL^{-1})	**EQM (%)** (7.45 ng mL^{-1})
**Mussels**	/	/
**Gastropods**	96	98
**Echinoderms**	95	94
**Arthropods**	96	94

EQM% = area of matrix-matched standard/ area of mussel-matched standard *100.

Table S4. HILIC-MS/MS method performances obtained for TTX in the studied matrices at two spiking levels: concentrations (μg kg^{-1}), recovery (R%), mean concentration (μg kg^{-1}) and mean recovery (R%) with the corresponding standard deviation (SD), and the precision expressed as inter-day relative standard deviation (RSD_R%).

Mussels					***Echinoderms***				
	**8 μg kg^{-1}**		**22 μg kg^{-1}**			**8 μg kg^{-1}**		**22 μg kg^{-1}**	
N	*μg kg^{-1}*	*R %*	*μg kg^{-1}*	*R %*	N	*μg kg^{-1}*	*R %*	*μg kg^{-1}*	*R %*
1	5.8	72	20.8	95	*1*	4.7	59	18.2	84
2	5.1	64	18.4	84	*2*	4.9	62	20.6	95
3	4.9	62	23.1	105	*3*	5.0	62	18.9	88
4	6.8	86	23.1	106	*4*	5.3	66	16.7	76
5	6.4	81	22.3	102	*5*	5.1	64	17.6	80
6	6.2	79	18.5	85	*6*	4.9	60	18.5	85
7	6.0	76	20.1	93	*7*	5.0	62	20.2	93
8	6.0	75	21.4	99	*8*	4.8	60	18.5	85
9	6.7	84	17.2	78	*9*	4.9	61	18.8	86
10	5.9	74	19.7	91	*10*	5.1	64	18.5	84
11	5.3	66	16.1	74	*11*	5.1	65	17.8	82
12	7.4	93	19.7	91	*12*	5.2	65	19.1	88
mean	**6.0**	**76**	**20.0**	**92**	*mean*	**5.0**	**63**	**18.6**	**86**
SD	**0.7**	**9**	**2.2**	**10**	*SD*	**0.2**	**2**	**1.1**	**5**
RSD_R%	**12**	/	**11**	/	*RSD_R%*	**4**	/	**6**	/
Gastropods					***Arthropods***				
	**8 μg kg^{-1}**		**22 μg kg^{-1}**			**16 μg kg^{-1}**		**32 μg kg^{-1}**	
N	*μg kg^{-1}*	*R %*	*μg kg^{-1}*	*R %*	N	*μg kg^{-1}*	*R %*	*μg kg^{-1}*	*R %*
1	6.6	82	22	98	*1*	12.2	77	24.8	77
2	6.5	83	19	89	*2*	12.3	78	26.8	83
3	6.3	80	20	93	*3*	13.6	84	25.3	78
4	7.3	91	22	99	*4*	11.6	73	27.6	85
5	6.7	85	22	100	*5*	11.4	72	24.8	76
6	7.1	91	18	82	*6*	11.4	71	24.3	76
7	7.4	91	20	94	*7*	12.6	79	28.7	89
8	7.6	95	21	97	*8*	11.3	71	25.6	80
9	7.0	88	21	97	*9*	11.2	71	23.9	75
10	7.9	87	21	95	*10*	11.3	71	25.5	79
11	7.4	92	21	98	*11*	12.4	78	27.1	84
12	7.0	88	18	84	*12*	12.0	76	26.4	82
mean	**7.1**	**88**	**20.5**	**94**	*mean*	**12.0**	**75**	**25.9**	**80**
SD	**0.5**	**5**	**1.4**	**6**	*SD*	**0.7**	**4**	**1.4**	**4**
RSD_R%	**7**	/	**7**	/	*RSD_R%*	**6**	/	**6**	/

N= replicated analyses, R%= recovery %, SD= standard deviation, RSD_R%= inter-day relative standard deviation.
